# Identifying integration and differentiation in a Hospital’s logistical system: a social network analysis of a case study

**DOI:** 10.1186/s12913-020-05514-w

**Published:** 2020-09-11

**Authors:** Annelies van der Ham, Frits van Merode, Dirk Ruwaard, Arno van Raak

**Affiliations:** Department of Health Services Research, Care and Public Health Research Institute (CAPHRI), Faculty of Health, Medicine and Life Sciences, Maastricht University/Maastricht University Medical Centre+, P.O. Box 616, 6200 Maastricht, MD The Netherlands

**Keywords:** Hospital, Logistics, Integration, Differentiation, Social network analysis

## Abstract

**Background:**

Integration, the coordination and alignment of tasks, has been promoted widely in order to improve the performance of hospitals. Both organization theory and social network analysis offer perspectives on integration. This exploratory study research aims to understand how a hospital’s logistical system works, and in particular to what extent there is integration and differentiation. More specifically, it first describes how a hospital organizes logistical processes; second, it identifies the agents and the interactions for organizing logistical processes, and, third, it establishes the extent to which tasks are segmented into subsystems, which is referred to as differentiation, and whether these tasks are coordinated and aligned, thus achieving integration.

**Methods:**

The study is based on case study research carried out in a hospital in the Netherlands. All logistical tasks that are executed for surgery patients were studied. Using a mixed method, data were collected from the Hospital Information System (HIS), documentation, observations and interviews. These data were used to perform a social network analysis and calculate the network metrics of the hospital network.

**Results:**

This paper shows that 23 tasks are executed by 635 different agents who interact through 31,499 interaction links. The social network of the hospital demonstrates both integration and differentiation. The network appears to function differently from what is assumed in literature, as the network does not reflect the formal organizational structure of the hospital, and tasks are mainly executed across functional silos. Nurses and physicians perform integrative tasks and two agents who mainly coordinate the tasks in the network, have no hierarchical position towards other agents. The HIS does not seem to fulfill the interactional needs of agents.

**Conclusions:**

This exploratory study reveals the network structure of a hospital. The cross-functional collaboration, the integration found, and position of managers, coordinators, nurses and doctors suggests a possible gap between organizational perspectives on hospitals and reality. This research sets a basis for further research that should focus on the relation between network structure and performance, on how integration is achieved and in what way organization theory concepts and social network analysis could be used in conjunction with one another.

## Background

Literature in the field of health care calls for more integrative approaches to the logistical or operational system of hospitals [1–3]. Such an approach includes aligning activities and planning resources from the perspective of the total system, taking hospital-wide processes and resources into account [4]. This is considered important because of a widely felt need to improve the quality, accessibility and affordability of healthcare systems [5] and of hospitals in particular, given the fact that hospitals are a major cost item of the healthcare system [6]. There is a wide consensus in literature that an integrated perspective on hospitals, which is a central concept in supply chain management, and in lean and other operations management theories, can contribute to the improvement of hospital performance [4, 7–12]. Integrated hospitals plan patient processes and resources from the perspective of the total system [9]. A lack of integration is attributed to the functionalistic organization structure of medical disciplines and departments, often referred to as functional silos [1, 4, 8, 10]. Ludwig et al. found evidence that hospitals that perform well score high on cooperation, while efficient departments within a hospital don’t necessarily contribute to the hospital’s overall efficiency [8]. There are, however, a few studies that show system-wide performance improvement when adapting integrative practices such as lean [12]. In an earlier scoping study [13] we found that research on logistics in hospitals typically focuses on one specific logistical flow (patients, material or staff) or on specific departments, but not on a system-wide level. The fact that 106 logistical performance parameters were identified which were applied in 92 subsystems [13], illustrates the absence of a hospital-wide performance framework for logistics. In addition, De Vries and Huijsman [7] point out that little is known on how to achieve integration in healthcare settings, and that this may require a different approach than in other industries.

Both contingency theory and social network theory offer perspectives on integration that could be useful in further exploring integration in the logistical system of hospitals.

Lawrence and Lorsch, who made a major contribution to contingency theory, view organizations as open systems in which the behaviors of members are interrelated [14]. They state that not only is integration important, but also that differentiation is essential in order for integration to be effective [15]. They define integration as ‘achieving unity of effort among the various subsystems in the accomplishment of the organization’s task’. Differentiation refers to ‘the state of segmentation of the organizational system into subsystems’. Subsystems execute a part of the organization’s task and can develop particular attributes in relation to the requirements posed by the relevant external environment [15]. From this perspective, integration is not an absolute quality or ideal. The necessary degree of integration is determined by ‘the felt need for joint decision making’, which also depends on the organization’s specific circumstances. To what degree and in what way integration and differentiation are effective may even depend on the ‘unique characteristics of each type of network studied’ [16].

Research in the field of social network analysis also addresses integration. Several authors mention network metrics to indicate integration in organizations or networks, thereby often referring to coordination between people, groups or organizations [16–18]. Differentiation is also mentioned in literature pertaining to social network analysis, when referring to tasks being differentiated [16, 19], but there are no specific metrics used that refer directly to differentiation. In his book, Kilduff [17] states in a chapter on social network analysis that ‘we await a full-blown contingency theory analysis of how trust-based coordinating mechanisms facilitate differentiation and integration’. The fact that this theory doesn’t yet exist could be attributed to the widely reported ‘embryonic’ stage [18] of social network analysis, as shown by two literature reviews [20, 21]. At the same time, several studies view social network analysis as a promising method. Benham and Clancy [22] view social network analysis as a new and creative method that is required to meet the complex problems of leaders in modern healthcare organizations. In multiple promising, though mostly exploratory studies, a relation between network structure and the performance of healthcare organizations or networks has been reported, both in terms of quality of care as well as efficiency. For example, Provan and Sebastian [16] indicate that organizations perform more effectively when integration is established through small groups of highly connected agents, when agents are included in multiple groups. Haythornwaite [23] points out that groups with strong relationships facilitate information exchange. Several authors mention the utility of ‘brokers’ or ‘integrative devices’ that join groups which are disconnected [15–17, 23]. Various studies report tentative results in which a link is made between the network structure and performance parameters such as surgery lead time [24], hospitalization cost [19, 25], process efficiency [26], readmission rate [25] and patient quality and safety outcomes [27]. At the same time these studies are said to provide weak evidence, which is attributed to the fact that social network analysis is an upcoming method [21].

In short, both contingency theory and social network analysis provide useful concepts for addressing the issue of integration in the logistical system of hospitals, but this needs to be explored further. Before we are able to say anything on how integration and differentiation may improve the performance of hospitals, we first need to know how a hospital and in particular its logistical system works. We need to know what the tasks are, who executes these tasks, how all tasks are aligned and whether we see integration and differentiation in the hospital system. Accordingly, the general objective of this research is to understand how a hospital’s logistical system works and in particular to what extent there is integration and differentiation. Specific objectives for achieving the general objective are:

1. Identify the agents and the interactions between them for organizing logistical processes
2. Describe how the hospital organizes logistical processes
3. Identify integration and differentiation as they exist in the entire hospital network.

We believe that understanding how a hospital’s logistical system works is a necessary first step towards improving the functioning of the hospital system. In this study, the hospital logistics are described from a system-wide perspective using social network analysis. To the best of our knowledge, this type of study has not been done before. Therefore this study should be considered exploratory. For this purpose a case study was conducted in a general hospital in the Netherlands, in which a social network analysis of the hospital’s logistical system was performed.

In line with our objectives, we focus on the following three questions:

1. What are the tasks executed for hospital logistics and which agents execute these tasks?
2. Which agents interact in executing these tasks?
3. To what extent do we see integration and differentiation in the network?

## Methods

In this section we explain how and in what setting the study was performed, what data were collected and how they were analyzed through a social network analysis.

### Setting

The study is based on the case study research method devised by Yin [28]. We selected Slingeland Hospital for our study because it is a relatively small Dutch hospital, has well-reported performance and the circumstances were relatively stable, as no large scale transformation projects were taking place. Additional selection criteria were good access to people and data. As a result of the merger with the Queen Beatrix Hospital in Winterswijk in 2017, this hospital became part of the larger Santiz group, but it functions largely as an independent full service hospital. The logistical operations for surgery across facilities were not combined in any way at the time of our research.

Slingeland Hospital has around 1600 staff members, including 120 physicians and 426 nurses. It services around 200,000 people in the area, and has 350 beds, which is below the average number of 450 beds for hospitals [29]. Slingeland Hospital performs higher on most logistical indicators than the average Dutch hospital, according to a Dutch OTC benchmark [30]. With an average of 89% operating room utilization in 2016, Slingeland has higher operating room (OR) utilization than the 82% average of Dutch hospitals that participate in a national benchmark. For other parameters, such as lateness and average surgery time, Slingeland performs better than the average hospital in the Dutch benchmark.

### Study design

With regard to our first two objectives and research questions on the tasks that are executed for hospital logistics and the agents who interact in executing these tasks, data were collected from multiple sources and then analyzed through data triangulation following a mixed method approach. With regard to our third objective, to establish integration and differentiation, a social network analysis was performed [31]. This analysis reveals the structure of the hospital network; the metrics developed in social network analysis methodologies can indicate the degree of differentiation or integration.

Using a system-wide perspective, ideally we would describe the entire intra-organizational network of a hospital. However, given the exploratory nature of our research and to reduce complexity and increase feasibility, it was decided to focus on the social network that includes all agents of all departments that execute tasks for the benefit of surgery patients. This includes agents of outpatient departments, the preoperative screening department, the nursing departments, the Operating Theatre Complex, the Central Sterilization Unit and the holding and recovery areas. By including all departments that take part in organizing patient flows, material flows and staff flows, a large part of the hospital system was included, which is in line with a system-wide approach. Moreover, the network in place for surgery patients is important, given the fact that more than 60% of patients who are admitted to a hospital are treated in the operating theatre complex (OTC) [32] and the OTC accounts for more than 40% of a hospital’s total revenue, and a similar proportion of its total expenses [33]. We studied the entire intra-organizational network of the hospital, because internal agents are primarily responsible for organizing logistics for patients, thereby focusing on the integration and differentiation within the bounds of the hospital.

### Data collection and analysis

For establishing the tasks that are executed for hospital logistics (question 1) and which agents interact when executing these tasks (question 2), data were collected from four different sources: the Hospital Information System (HIS), documentation, observations and interviews. The data collection focused on identifying all tasks for surgery patients in 2017, including the interaction between the agents involved in these tasks. The collection and analysis of data from the HIS and documentation took place in January and February of 2018. Following that, observations took place between March and April of 2018. The findings from the data and from the observations resulted in some knowledge gaps, which were further explored in 11 interviews; these took place between May and September 2018.

The HIS data include registrations of surgeries performed in 2017, including date of surgery, staff involved, materials used and timestamps of different stages of the surgery patient’s process, as well as of the nursing wards the patients were in before and after surgery. Other data of the HIS include, for example, the number of staff members and the planning schedules.

Documentation includes planning schemes, working procedures and internal presentations on internal processes which were valid at the time these were collected, at the start of 2018.

The daily work of 12 departments was observed on 14 different days. The observations took place in three outpatient departments, at the preoperative screening department, in two nursing departments, in the OTC and the Central Sterilization Unit and in the holding and recovery areas. The departments were selected because they execute tasks that contribute to the overall task of performing surgery. A total of 98 people were observed, including both staff and patients. Observations were conducted using a naturalistic approach, as described by Beuving and De Vries [34]. Each observation day was prepared by studying HIS data and working procedures in that department on the day before the observation took place. These data were used to formulate broad questions for the observer to keep in mind during the observation. In order to avoid creating a formal setting, the observer had casual conversations with the observed staff only when staff initiated this and if the observer felt that this contributed to keeping the situation natural. The observer made field notes of events including timestamps, conversations and observed behaviour, which were reported in an observation report.

For the interviews we selected people of different agent types that we had met during the observations, but with whom we had not spoken comprehensively, and we also selected people who were suggested to us by hospital staff whom we had met during data collection and observations. Two surgeons, one anesthesiologist, the cluster manager for the OTC and Services, two OTC team leaders, the OTC capacity planner, a business controller and the application controller were interviewed. For each interview a topic list was prepared; topics include the logistical tasks and interaction with other agents, which demands the agent has to deal with in relation to these other agents and how the network functions as a whole. All interviews were recorded and transcribed ad verbatim.

With these data, the logistical tasks that are executed for patients who have surgery and the order in which they are executed were identified. A task is seen as a ‘complete input-transformation-output cycle’ [15] for a particular intended result. The focus was on tasks which are triggered directly by the patient and for which interaction between agents was found. Interaction includes face-to-face contact or communication via telephone, email or text messages. Tasks relating to small surgeries that are performed in the outpatient departments were excluded.

Each source was used to identify the interaction relations between the agents involved in each task. For this, the data from the four sources, both quantitative and qualitative, were combined using data triangulation [28]. Interactions were first of all directly derived from HIS data; for example, who was involved in each surgery in 2018 is registered in the system, and it was observed that these agents interact during the surgery. Interactions were also derived from standard working procedures, which were both described and observed; these are interactions as they generally take place between agents with the same function or role. In addition, interviewees were asked specific questions on which agents interact with whom for task execution. In most cases the interactions were derived by combining data from all sources. For example, in the observations we saw that the surgeon visits the nursing ward to see his patients. In the interviews the surgeon explained that this is a daily activity and that he then always interacts with a ward nurse. From the HIS data it was derived how many surgeons and ward nurses there are and on which ward the patients of each surgeon were located. Additional file 1 shows which sources provided the input for establishing the interactions for each task.

For each task the working procedures and interactions were first described in text and then the interaction for task N between agent A and B, B and C and so on were registered in an Excel database. Each agent was anonymized by using a code that consists of three letters of either the department or the medical discipline the agent works for and an abbreviation of the function of the agent and a number. A Urology surgeon is UROS1 with URO being the medical discipline, S for surgeon and 1 for the specific agent. This resulted in a structured database of 39,055 rows. Each line in the database represents an undirected communication link – a tie - between agents A and B for a specific task N. Each node in the network represents an agent, who is an individual person. Because this study focuses on identifying integration and differentiation by analyzing the social network structure, the interaction frequency was not included in the research and the ties do not have any weight. All interactions described are a result of working procedures and common ways of repetitively executing tasks throughout the year.

Having established the tasks, agents and interaction, a social network analysis was performed in order to elicit integration and differentiation (question 3). The social network was built up from the identified interactions between agents per task, as recorded in the database. The database was inserted in NodeXL [35], which was used to construct the social network, The Harel Koren Fast Multiscale Algorithm was used for structuring the network. This algorithm was developed specifically for the fast and clear visualization of large social networks [36]. It structures the network in such a way that agents who are linked and have similar links to other agents are positioned close to each other in the network. In addition, agents with a relatively high number of ties in comparison with other agents are positioned in the center of the network.

Specific concepts and measures of the social network that are related to the concepts of integration and differentiation were analyzed. In line with Provan [16], Kilduff [17] and Haythornwaite [23], density, degree, betweenness centrality and clique overlap were used as indications for integration. No metrics were found for differentiation in social network literature, but Kilduff [17] and Monge [18] associate differentiation with the existence of groups or cliques that consist of highly connected agents. There is a clique when all agents in a group are connected. These measures are presented in Table 1.

**Table 1:** Definition of network concepts and metrics

Concept	Definition
Node	An agent
Tie	A communication link between two agents via email, text message, telephone or face-to-face
Group	A set of agents who are closely connected to one another
Clique	A set of agents who are all connected to one another
Subsystem	A set of agents who are highly connected and execute a part of the organization’s overall task
Broker	An agent who connects (otherwise) disconnected groups
Density	The number of ties a set of agents have in relation to the number of possible ties they can have
Clique overlap	The percentage of agents who are members of more than one clique for a specific task
Degree	The number of ties of one agent
Betweenness centrality	The number of times a node (agent) lies on the shortest path between other nodes (agents)
Multiplexity	The percentage of agents in a clique for a task who are also members of cliques for other tasks.
Centralization	The extent to which a set of nodes (agents) are organized around a central node (agent)

On a network level, the entire hospital network was analyzed to identify groups of densely connected agents. A group consists of highly connected agents in which case there is high density. Density.

is defined as size relative to the number of possible ties and calculated by the ratio of the number of actual links between nodes and the maximum possible edges for the network [31]. A relatively low density for the entire network suggests differentiation or, put the other way around, a lack of integration.

In addition to density we also looked at clique overlap and at multiplexity for integration in the entire network. There is clique overlap when agents are part of more than one clique, thereby connecting different cliques [16, 17]. Clique overlap was calculated by dividing the number of agents participating in multiple cliques by the total number of agents. When there is clique overlap across different tasks, there is multiplexity [16]. Multiplexity is the percentage of agents in a clique for a task who are also members of cliques for any other task.

The clique analysis per task was performed in order to see how the organizational system is segmented into subsystems, following Lawrence and Lorsch’s definition of subsystems [15]. Breaking down the structure of the overall task of the hospital network into smaller tasks reveals in what way tasks are differentiated. This was done by filtering the database by task and analyzing this part of the network in NodeXL accordingly. Cliques were also identified for each task, revealing possible smaller subsystems.

We looked at betweenness centrality for agents who act as a broker [16, 17] in the network. According to Haythornthwaite [23] brokers are ‘connections between disorganized others’ and they carry information from one group to another. Agents with a high betweenness centrality have an intermediary position between others in the network [23, 31]. This metric represents the number of times a node lies on the shortest path between two other nodes [37] and was calculated with the algorithm used in NodeXL [38].

**Fig. 1:**
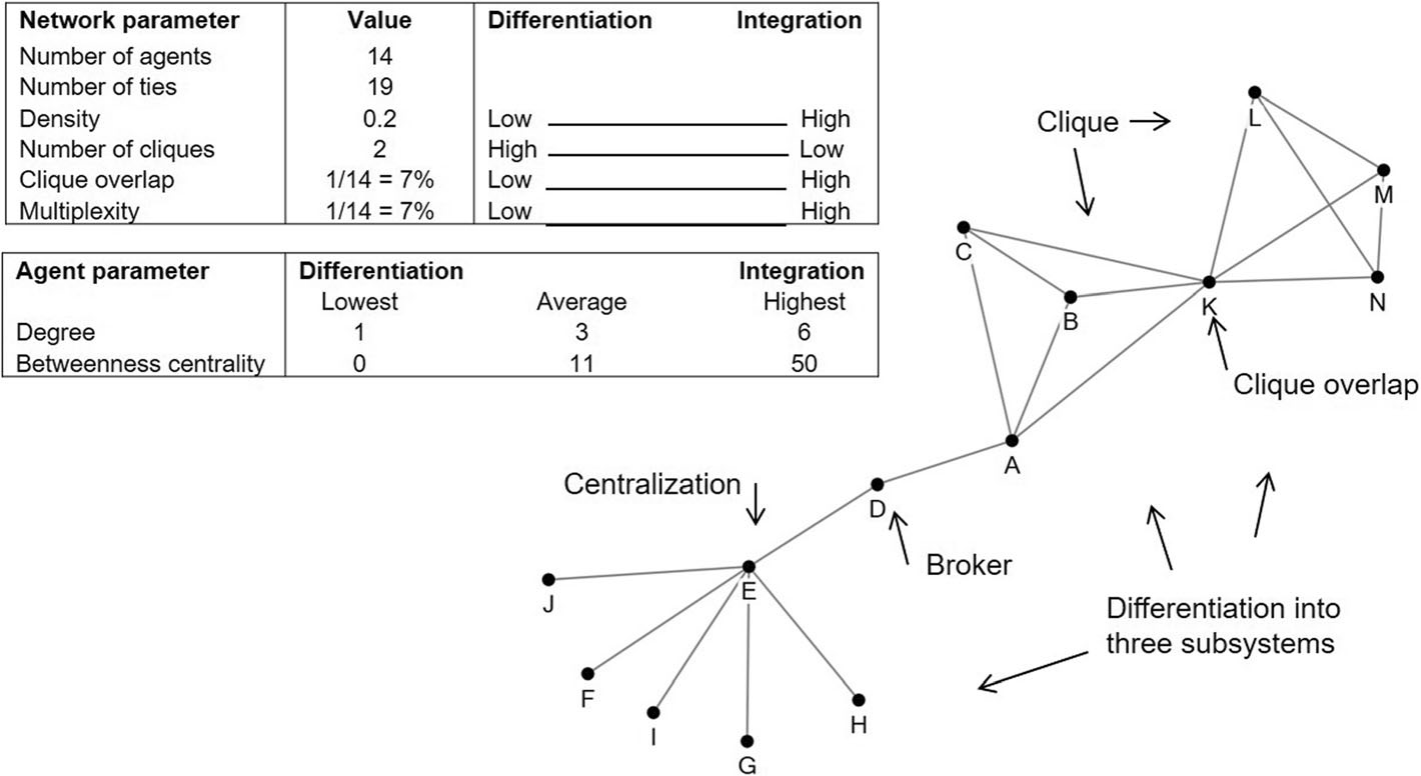
Example network with SNA concepts and metrics

Further, we looked at centralization, which is defined as ‘the extent to which a set of actors are organized around a central point’ [23]. In a centralized network there is a high standard deviation in the degree of agents, i.e., in the number of ties, because some agents have a high degree and most others have a low degree [39]. Centralization may suggest differentiation, as agents around the central agents could be isolated from the rest of the network, as is the case for nodes F to J in the example presented in Fig. 1. It is important to note that centralization in social network analysis is different from the widely accepted definition of Mintzberg, who states that centralization is related to decision- making power [40].

In Fig. 1 an example of the social network analysis is presented, including the metrics.

### Network validation

For validation purposes, the social network per task was discussed face-to-face between August 2018 and November 2018 with 10 hospital staff members who were also involved in the observations and interviews. Specific details were asked via email to specific members of hospital staff who perform the tasks identified. Remarks from hospital staff were reported in a validation report.

## Results

All tasks and interaction taking place between agents in executing these tasks are described in this section. Further, the entire hospital network, which is built up by these interactions, is presented and integration and differentiation are described. We start this section with some key figures on surgeries in Slingeland Hospital.

### Output of the social network

In 2017, 10,157 surgeries were performed in Slingeland Hospital. The number of surgeries varies from a minimum of 4 to a maximum of 246 surgeries a week. Of all surgeries, 83% are planned beforehand, i.e., they are not emergency procedures. Different types of surgeries are performed, which are registered according to 394 treatment codes in the HIS. These treatment codes are divided among nine medical disciplines: general surgery, orthopedics, Ear Nose Throat (ENT) surgery, eye surgery, urology, gynecology, plastic surgery, dental surgery and neurosurgery. Of all 394 treatment codes, on average 66% are performed once a month or less and 9% are performed on a weekly basis. More than half of the treatment codes are executed by only one or two specific surgeons. For example, 103 treatment codes are performed by one specific, but not the same, surgeon. For 42% of all surgeries, there was a unique one time combination of treatment code, surgeon and anesthesiologist. This and the fact that in 2017 a total of 2881 unique combinations of medical instrument sets were used, suggest that human and material resources are not fit for a large variety of surgeries, but are mostly suitable for specific surgeries.

### Agents and tasks performed for surgery

**Fig. 2:**
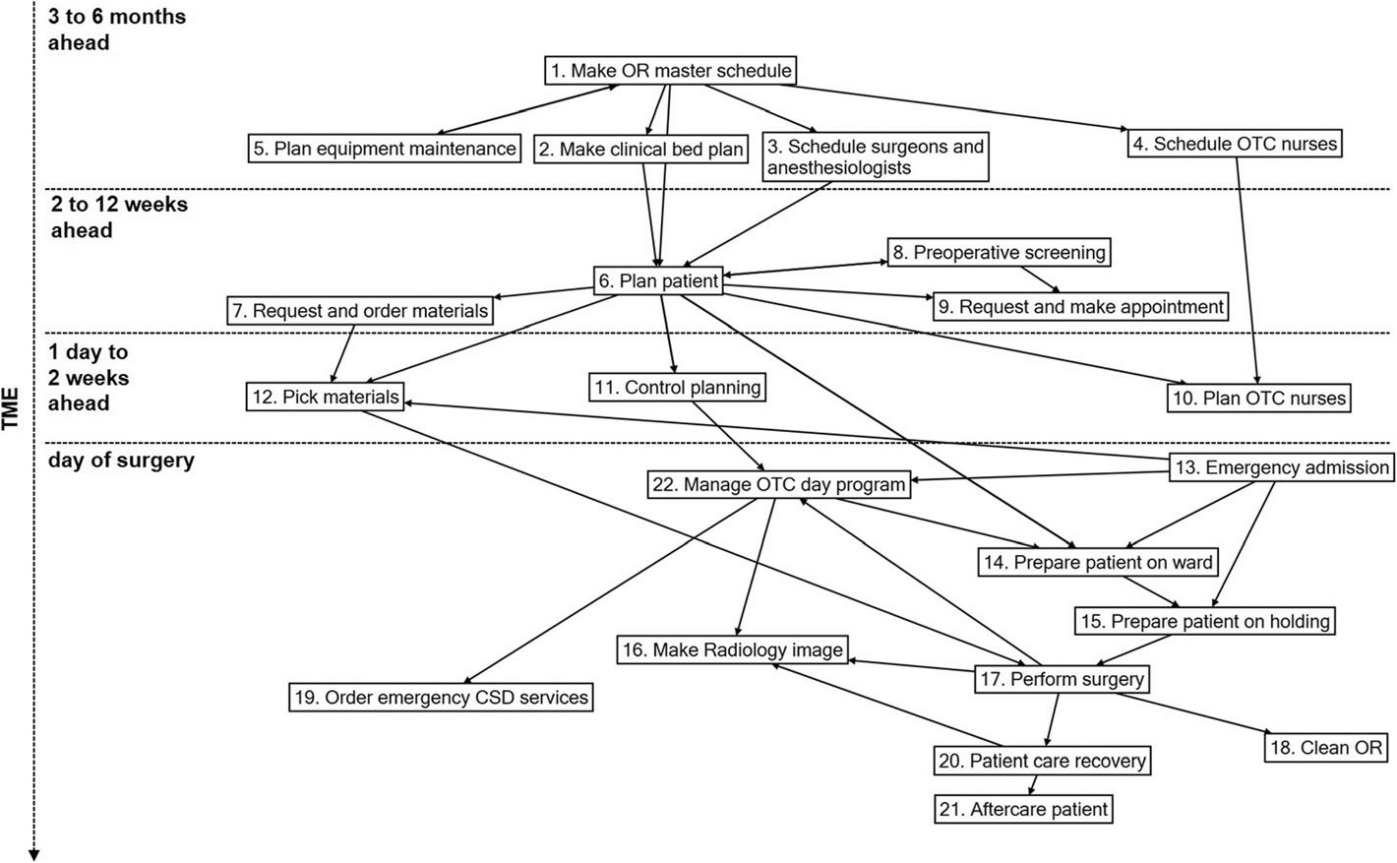
The links between tasks performed for patient surgery

The main task of the logistical system is to get the right patient, surgeon, anesthesiologist, nurses, materials and infrastructure together at the right time and in the right place. There are 23 tasks that are executed in order to achieve this, as presented in Table 2. Figure 2 shows the relation between 22 tasks, mostly based on the chronological order in which these are executed. In addition, the arrow between two tasks means that the output of a task is input to the task to which it is connected. Task 23, managing the OTC, is not specifically time dependent, nor is there specific output of this task and therefore it is not mentioned in Fig. 2. Tasks 1 to 5 are at the tactical level because these concern master scheduling in the medium-term [41]. The other tasks are operational because they are related to short-term allocation of resources and execution. Long-term strategic tasks such as demand forecasting were found, but these do not relate directly to the tasks shown in Fig. 2. Overall, two main groups of tasks are visible in Fig. 2: tactical and operational planning 6 months ahead until the day before surgery and the execution of the surgery process.

**Table 2:** Tasks and agent types involved in these tasks

	Tasks	MSC	OTC	MSC	Cluster OTC and Services	CSD	ER	ER	MIS	OTC	OTC	OTC	OTC	OTC	OTC	OTC	OTC	Outpatient dpt (5)	Pharmacy dpt	Preop dpt	Preop dpt	Radiology dpt	OTC	MSB	Nursing ward (7)	Nursing ward A2	Nursing ward (7)	Task description
		Anesthesiologist	Nurse anesthetist	Assistant surgeon	Cluster manager	CSD staff member	ER physician	ER nurse	Equipment maintenance staff	Holding nurse	OTC capacity planner	OTC cleaning staff	OTC day coordinator	OTC Logistical staff	OR nurse	OTC secretary	OTC team leader	Outpatient secretary	Pharmacy assistant	Preoperative nurse	Preoperative secretary	Radiology staff	Recovery nurse	Surgeon	Ward nurse	Clinical bed plan boss	Ward team leader	
1	Make OR master schedule	x			x						x							x						x				Make the OR master schedule in which operating time for each medical discipline is allocated to the operating rooms for even and uneven weeks.
2	Make clinical bed plan										x															x	x	Make the clinical bed plan in which beds are allocated to medical disciplines per nursing ward for even and uneven weeks.
3	Schedule surgeons and anesthesiologists	x																x						x				Determine the working hours for every surgeon and anesthesiologist for the upcoming three to six months, including where they are working, i.e., in the outpatient department and the OTC.
4	Schedule OTC nurses		X							x			x		X		x						x					Determine the working hours for every nurse anesthetist, OR, holding and recovery nurse for the upcoming three to six months.
5	Plan equipment maintenance								x		x		x				x											Determine which OTC equipment will be maintained on what day and time and how long the equipment will be unavailable for use.
6	Plan surgery										x							x						x		x		Determine the time and date that the patient will be operated on and register this in the OR master schedule.
7	Order materials												x		X			x						x				Request specific materials for one specific surgery and order these materials at external supplier(s).
8	Preoperative screening	x																	x	x	x							Determine what type of anesthetic technique fits the patient, what potential risks should be considered during the patient’s surgery, and what is the best preparation for the surgery of this patient.
9	Make appointment	x																x			x							Request visits to physicians, laboratory or radiology tests for preparation of the patient for the surgery.
10	Plan OTC nurses		X							x			x		X								x					Determine the working hours for every nurse anesthetist, OR, holding and recovery nurse for the upcoming week, including what surgeries they assist.
11	Control planning										x		x			x	x	x			x				x			Check all requirements for the surgery to be able take place, determine the final order of the surgeries for each OR and if necessary revise planned surgeries.
12	Pick materials										x		x	x	X													Collect materials required for surgeries from the storage rooms and deliver these to the operating rooms.
13	Emergency admission	x		x			x	x					x											x	x			Define diagnosis and treatment for patients admitted to the Emergency Department and plan and prepare the patient for surgery.
14	Prepare patient on ward		X																						x		x	Admit the patient to the nursing ward, administer premedication to the patient and further prepare the patient for surgery.
15	Prepare patient in holding		X							x															x			Transfer the patient from the nursing ward to holding, further prepare the patient for surgery and transfer the patient to the nurse anesthetist.
16	Make Radiology image												x		X	x						x	x					Ask the radiology department to make an image of the patient in a specific place (OR or recovery) and time.
17	Perform surgery	x	X	x											X									x				Perform the surgery with the OR team.
18	Clean OR											x			X													Ask the cleaning services to clean the operating room right after transferring the patient to recovery.
19	Order emergency CSD services					x							x															Call the central sterilization department to ask for immediate cleaning and sterilization of medical instruments, as these may be re-used for surgery on the same day.
20	Patient care recovery	x	X																				x		x			Take care of the patient after surgery, making sure the patient is well enough to be transferred to the nursing ward.
21	Aftercare of patient																							x	x		x	Take care of the patient after surgery, making sure the patient is well enough to go home.
22	Manage OTC day program	x	X							x	x		x		X		x	x					x	x				Coordinate and manage the daily OR program, making sure that all surgeries planned for each day are well-executed and on time, and review this over time.
23	Manage OTC tasks				x						x		x				x											Coordinate and manage OTC operations over the long term.
	**Total number of tasks involved**	8	7	2	2	1	1	1	1	4	8	1	11	1	8	2	5	7	1	1	3	1	5	8	6	2	3	

*MSC* Medical Specialty Company, *CSD* Central Sterilization Department, *MIS* Medical Instrumental Services, *OTC* Operating Theatre Complex, *ER* Emergency Room department, *Preop dpt.* Preoperative Screening department, *(n)* number of departments

First the OR master schedule is made for a three-month period (task 1), two quarters ahead; the OR master schedule for Q2 of any year is made in Q4 of the previous year. In the OR master schedule, time slots for all ORs are allocated to the nine medical disciplines that operate in the OTC. The clinical bed plan (task 2), equipment maintenance planning (task 5) and staff schedules (tasks 3 and 4) are all derived from the OR master schedule. Around 2 to 12 weeks before surgery, patients are planned into the OR program (task 6) and preparations start: patients are screened by an anesthesiologist (task 8), materials are ordered (task 7) and patients are seen by other physicians or take radiology or laboratory tests (task 9). In the days before surgery further preparations are made: the OR day program is planned in more detail (task 11), staff is allocated to specific surgeries (task 10) and materials are picked (task 12). On the day of surgery the patient is prepared and held on the ward (task 14) before the actual surgery takes place (task 17) and is afterwards taken care of in the recovery area (task 20) and ward (task 21). In some cases a radiology image is made during or after surgery (task 16). After surgery the OR is cleaned (task 18) and if necessary the medical instruments are immediately cleaned for reuse (task 19). Patients can also be admitted for an emergency surgery (task 13), in which case all tasks are executed within a short period of time. All tasks have been specified in more detail in Additional file 2.

Task 23 is not included because it is not time-related to one of the other tasks.

Tasks are related to patient, staff and material flows. Tasks 5, 7, 12, 18 and 19 are related to materials and tasks 1, 3, 4 and 10 are about staff flows. Tasks 2, 8, 9, 13 and 16 are related to patient flow. The other tasks are related to more than one flow; for example, preparing a patient on the ward before surgery involves both the patient and medication.

For each task a number of agent types is involved, as presented in Table 2. The OTC day coordinator participates in 11 of the tasks and has the highest involvement in multiple tasks. The OTC capacity planner, anesthesiologists, surgeons and the OTC nurses all participate in eight different tasks. The other agents participate in fewer tasks, with a minimum of one. The task with the most different agent types involved is managing the OTC day program; 10 different agent types play a role in this.

With regard to the flows, most agents are involved in tasks related to patients, staff and materials. The CSD staff members, equipment maintenance staff, OTC cleaning staff and the OTC logistical staff are the only agents who deal with just one flow type, which is materials.

Table 2 shows that a number of tasks have an overlap in types of participating agents. This is particularly relevant when tasks are related. For example, tasks 2, 3, 4 and 5 are all related to task 1, and there is overlap in agent participation for the OTC capacity planner for tasks 1 and 2. The outpatient secretary, the anesthesiologist and the surgeon are all involved in both tasks 1 and 3. There are no overlapping agents for related tasks 1 and 4, 6 and 8, 6 and 14, 13 and 14 and 13 and 15. For tasks 4, 8 and 14 relevant information resulting from tasks 1 and 6 are communicated through the HIS, which then is the only information source for agents executing these tasks. For all other related pairs of tasks there are agents who participate in both tasks.

### The entire social network

**Fig. 3:**
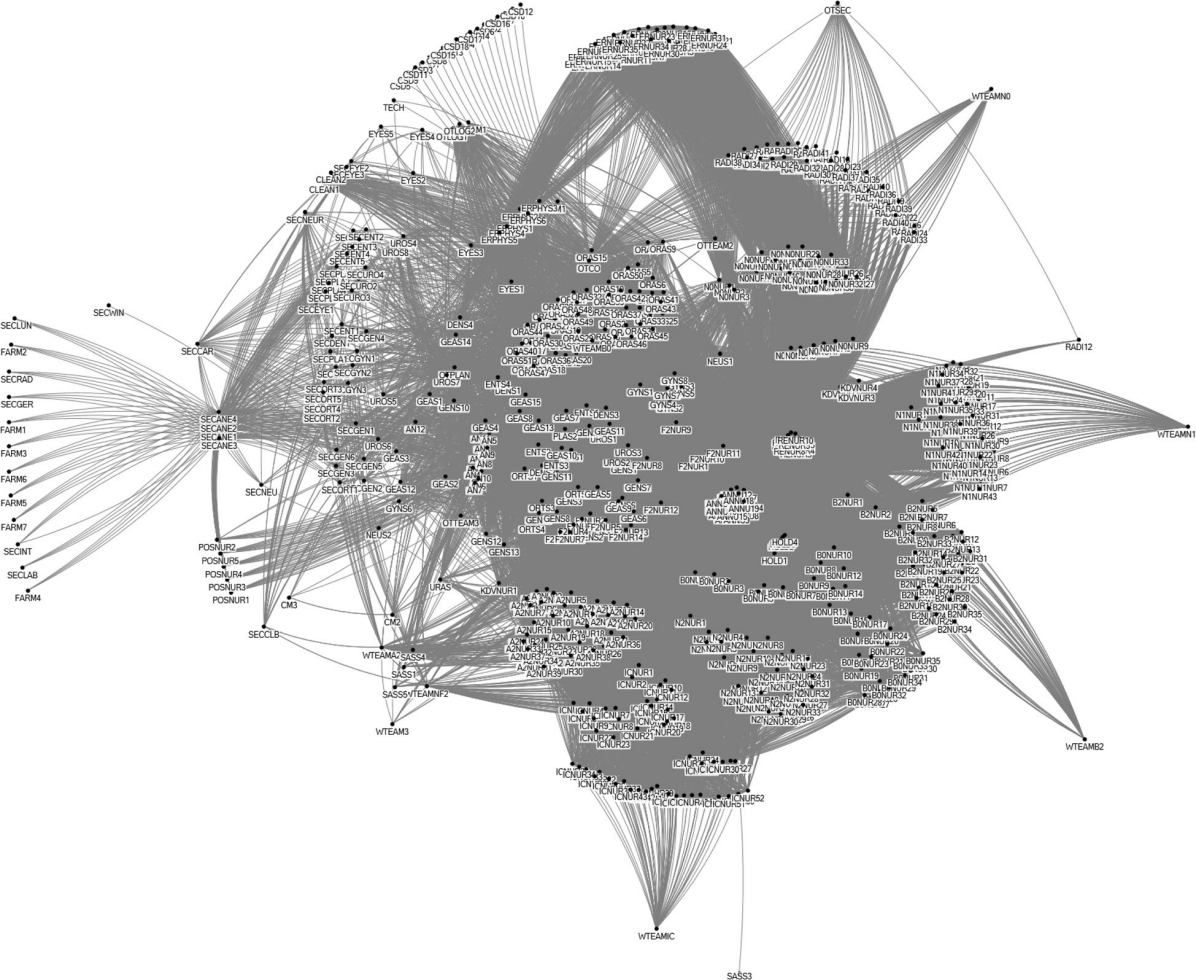
The social network of hospital logistics for surgery patients

Figure 3 shows the entire social network with all agents and the ties between these agents. The names of all agents were abbreviated in the network figures and are explained in Additional file 3. Even though Fig. 3 does not reveal the details of the network, it clearly shows that all agents are connected in one way or another and that there are no agents or cliques that are completely disconnected from the rest of the network. The relatively low density of 0.16, as shown in Table 3, indicates that there are agents or groups which are less connected, suggesting differentiation. The high number of cliques also indicates the presence of subsystems, demonstrating differentiation. However, 65% of all agents are part of multiple cliques across two related tasks. This high multiplexity value implies that there is integration as well. The spread between average and highest values for degree and betweenness centrality suggest that a relatively small number agents play an integrative role.

**Table 3:** Network metrics overall network

**Network Parameter**	**Value**		
Number of agents	635		
Number of ties	31,499		
Density	0.16		
Number of cliques	8698		
Multiplexity	413/635 = 65%		
**Agent parameter**	**Lowest**	**Average**	**Highest**
Degree	1	99	399
Betweenness centrality	0	347	31,379

Figure 3 also shows groups of agents who are closely connected, which suggests the presence of subsystems. We see groups of agents who share the same task or knowledge, or they deal with a specific patient group depending on age, condition or required length of stay. Examples of agents sharing the same task and patient group are on the top side edges of the network where we see the ER nurses and on the right side the nursing wards, which are all cliques; clockwise the groups of nurses are visible with codes KDVNUR, N2NUR, B0NUR, N1NUR, N0NUR, B2NUR, A2NUR. Each code starts with the name of the nursing department as defined by Slingeland Hospital, e.g., KDVNUR1, KDVNUR2 et cetera are nurses from department KDV. They also form subsystems because these nurses are all involved in the same task. Interestingly, the team leaders (WTEAM) of nursing wards B0, N1, N0, B2, IC have fewer connections to others in the hospital in comparison with the nurses, illustrated by their peripheral position in the network.

The group of intensive care (IC) nurses (ICNUR) form a clique as well, but they are more centrally positioned. This is because IC nurses have connections to all other nursing wards, as IC patients are always transferred to another nursing ward before they are discharged. Agents working in the daycare department F2, where patients stay because of their expected one-day length of stay, are also more centrally located because patients are transferred in case they need to stay the night.

All OR nurses (ORAS) also form a group, in three cliques, because they are divided into three clusters which are based on shared knowledge of medical disciplines. The holding and recovery nurses each have a clique as well. The anesthesiologists (AN) are visible as a group as well as the nurse anesthetists (ANNU), who are in the middle of the network. The surgeons do not form one group, but they form nine cliques that each share the knowledge of a specific medical discipline. Here we see separate subsystems according to medical discipline, which essentially all perform the same task. This is also the case for the secretaries of the outpatient departments, who are visible in the bottom left part of Fig. 3.

The high number of cliques is largely explained by the fact that there are 7640 unique cliques that perform surgery (task 17). This will be analyzed further in the network analysis of each task.

The legend for the agent codes is included in Additional file 3.

The average degree is 99 and standard deviation is 79, which suggests centralization, as there are relatively large differences between the number of ties of agents. The agent with the highest degree is a nurse anesthetist with 399 ties to other agents. The nurse anesthetists all have a high degree, with an average of 387 ties. On the day of surgery they have interaction with all surgery team members, including surgeons, anesthesiologists and OR nurses. Furthermore, they interact with all ward nurses, and with holding and recovery nurses throughout the year. This is also the case for holding and recovery nurses who have an average degree of 300 and 318, respectively. The agents with relatively low degrees are on the edges of the network in Fig. 3, e.g., all staff from the Central Sterilization Department (CSD) on the top left. In Additional file 3 the degrees of all agents are presented.

The OTC day coordinator (OTCO) has the highest betweenness centrality, which makes sense given the name of that function, but at the same time it is striking, because he does not contribute to multiplexity. The OTC capacity planner has the second highest centrality, and she has a strong integrative role between related tasks. The nurse anesthetists (ANNU) have high betweenness centrality as well as having a high degree, which also suggests a broker role.

The number of agents and communication links between them are different in the four time horizons which were presented in Fig. 2. Table 4 clearly shows that the number of agents interacting and the density is higher on the day of surgery than before that day. If we look at the planning and execution phase, the density is 0.08 and 0.16 respectively. This suggests that, even though the overall network integration is low, in the months, weeks and days before surgery there is more differentiation and less integration than there is on the day of surgery. Furthermore, the OTC capacity planner plays a more prominent role before the day of surgery, whereas the OTC day coordinator is mainly involved on the day of surgery.

**Table 4:** Network metrics of the network over time

Time horizon	Number of agents	Number of ties	Density	Highest betweenness centrality
3–6 months	168	1041	0.07	OTPLAN
2–12 weeks	146	695	0.07	OTPLAN
1 day to 2 weeks	144	428	0.04	OTPLAN
Day of surgery	605	30,135	0.16	OTCO

In the next section we will go into more detail of the network for each task.

### Network analysis per task

The social network per task is included in Figures 1 to 23 in Additional file 2. Table 5 shows the differences in network metrics between tasks. The number of participating agents varies from 4 to 391, the density from a low 0.01 to the maximum of 1, the number of cliques varies from zero to 7640 and clique overlap is between zero and 92%.

**Table 5:** Network metrics for each task

	Tasks	Number of agents	Number of ties	Density	Number of cliques	Clique overlap		Organization unit
1	Make OR master schedule	28	110	0.3	2	6	21%	Cross functional
2	Make clinical bed plan	4	6	1.0	1	N/A		Cross functional
3	Schedule surgeons and anesthesiologists	70	206	0.09	10	0	0%	Cross functional
4	Schedule OTC nurses	88	801	0.2	6	1	1%	OTC
5	Plan equipment maintenance	4	6	1.0	1	N/A		Cross functional
6	Plan patient	92	315	0.08	48	1	1%	Cross functional
7	Request and order materials	61	139	0.08	0	N/A		Cross functional
8	Pre-operative screening	27	109	0.31	12	9	33%	Cross functional
9	Request and make appointment	56	140	0.09	0	N/A		Cross functional
10	Plan OTC nurses	85	84	0.02	0	N/A		OTC
11	Control planning	54	234	0.16	2	3	6%	Cross functional
12	Pick materials	55	107	0.07	53	2	4%	OTC
13	Emergency admission	139	2840	0.30	1	N/A		Cross functional
14	Prepare patient on ward	314	11,071	0.23	171	19	6%	Cross functional
15	Prepare patient on holding	289	1100	0.03	285	4	1%	Cross functional
16	Make radiology image	53	491	0.36	0	N/A		Cross functional
17	Perform surgery	148	5444	0.50	7640	136	92%	Cross functional
18	Clean OR	53	102	0.07	0	N/A		OTC
19	Order emergency CSD services	19	18	0.11	0	N/A		Cross functional
20	Patient care recovery	241	2355	0.08	285	10	4%	Cross functional
21	Aftercare of patient	391	12,537	0.16	178	266	68%	Cross functional
22	Manage OTC day program	184	189	0.01	1			OTC
23	Manage OTC tasks	6	14	0.93	2	4	67%	OTC

Tasks with a relatively low density suggest differentiation. In Additional file 2 we see two network structures for such tasks: a network with weakly connected or disconnected cliques and a network with centralization. Task 3 (Figure 3 in Additional file 2) is a clear example of a network with 10 cliques that are all disconnected. Here we see differentiation according to medical discipline with regard to how surgeons and anesthesiologists are scheduled. Each medical discipline represents a subsystem. This is also the case for task 6, but here the medical disciplines are situated around the OTC capacity planner in a star network.

Other tasks with a centralized network are 7, 9, 10, 12, 15, 18, 19, 20 and 22. The centralization is first explained by the fact that tasks are coordinated by the OTC day coordinator (tasks 7, 10, 19, 22). For the other tasks there is centralization because the central agent in each network interacts with each agent individually, while these agents do not interact with one another for that task. For example, on a regular basis the two logistical staff members ask all OR nurses, the OTC day coordinator and the OTC capacity planner for information on surgeries for which they pick the materials. Based on the definition of subsystems, these star networks do not have subsystems, because the agents are not highly connected.

Interestingly, the central agents in these star networks do not have a hierarchical position towards the agents around them, because the networks are cross functional (tasks 6, 7, 15, 19, 20). For tasks 4, 10, 12, 18 and 22 the central agents do not have a formal hierarchical position towards the other agents either.

For tasks with a higher density such as tasks 1, 2, 5, 8 and 11 we see integration, either by the presence of one clique (tasks 2 and 5) or multiple cliques (tasks 1, 8, 11). Furthermore, we see a network for task 14 with a highly connected group or subsystem with multiple agents in the center (Figure 14 in Additional file 2). Doing surgery in the OR (task 17) looks like a cloud of connections (Figure 17 in Additional file 2) because surgeons, anesthesiologists, OR nurses and nurse anesthetists work together in 7640 different cliques.

Besides density, clique overlap is an indication of integration. For tasks 1, 8, 17, 21 and 23 there is a relatively high overlap of 33% up to 92%, but for the other tasks clique overlap has a maximum of 6%, in which case the integration depends on just a few agents.

Remarkably, almost all tasks in which integration is observed are all organized in a cross functional manner. Managing the OTC tasks is the exception here, as this is done by agents who work only for the OTC department.

With regard to betweenness centrality per task, different agents act as a broker. The OTC capacity planner is most central for making the OR master schedule and planning surgeries. The OTC day coordinator is most central for scheduling and planning OTC nurses, for ordering materials and for responding to emergency orders from the CSD. For other tasks the agents with the highest betweenness centrality are two surgeons (task 3), the preoperative nurses and secretaries (task 8), the Neurology and Cardiology nurses (task 9), OTC logistical staff (task 12), the nurse anesthetists (task 14), holding nurses (task 15), one OR nurse (task 17), cleaning staff (task 18) and recovery nurses (task 20).

If we consider the four time periods of Fig. 2 we see that the values for numbers of cliques and clique overlap are significantly higher on the day of surgery than for before that. This suggests that there is more differentiation as well as integration on the day of surgery than in the phases before. The high number of cliques is explained mainly by the fact that teams often interact for one specific patient; because these teams change frequently throughout the year, this results in a high clique overlap. In contrast, in the first phase, 6 to 3 months before surgery, there is a permanent smaller set of agents who make the OR master schedule, the clinical bed plan and equipment maintenance plan. Here we mainly see integration and no differentiation. At the same time the scheduling of surgeons and anesthesiologists (task 3) is executed by disconnected cliques, which shows differentiation.

## Discussion

This study aims to understand how a hospital’s logistical system works and in particular to what extent there is integration and differentiation. The three specific objectives are (1) to identify the tasks that are performed in arranging the logistics for a hospital’s surgery patients and (2) to establish which agents involved in these tasks interact with each other. In addition, (3) the degree of integration and differentiation in the entire hospital network is established. In total, 23 logistical tasks that are executed in-hospital for surgery patients have been identified. Twelve tasks are related to planning and 11 tasks are performed in executing surgeries by 635 different agents of 26 different agent types. The social network analysis shows that in the execution of these tasks there are 31,499 ties between these agents representing social interaction.

In the entire social network of the hospital both integration and differentiation are observed. The overall hospital network has a relatively low network integration, according to the low density, and there is differentiation in the execution of tasks per medical discipline, organizational unit and cross functional groups. Despite the overall low network degree, integration is demonstrated in cliques, in high clique overlap for several tasks and in multiplexity.

In contrast to the literature, which states that tasks are performed within functional silos [1, 4], this study shows that most tasks in the case are executed across functional silos. Agents are involved in many different tasks, which are related to patient, material and staff flows. Apart from the way patients are admitted, there is no difference in tasks and involved agents between emergency and planned surgeries. There are several agents who act as a broker, but the OTC day coordinator and the OTC capacity planner are the only two agents whose primary task is to perform typical broker tasks such as network coordination and planning. Their betweenness centrality is substantially higher than for other agents. Besides these two agents, many nurses are brokers, as demonstrated by the relatively high degree and betweenness centrality of the nurse anesthetist, OR, holding, recovery and ward nurses. Other agents who integrate tasks are surgeons and anesthesiologists.

The social network analysis also demonstrates that agents with management roles, such as the cluster manager of the OTC and Services or team leaders, have a relatively low degree and betweenness centrality. Even in networks with centralization, the position of central agents is not based on hierarchy or formalized decision power. Almost all central agents in the networks per task have no hierarchical position with regard to other agents.

These findings suggest that the hospital’s logistical system works differently than what is assumed in logistical and organizational literature with regard to hospitals. The network of Slingeland Hospital does not reflect its formal organizational structure. Nevertheless, interestingly enough, when we look at the hospital’s performance, the system as described seems to function.

One explanation might be that in research, informal processes or interaction are not included, even though these take place in practice, due to the high variation in patient demand and uncertainty in the system. We could also argue that the hospital’s logistical network is a relatively independent system, in which patient care is exclusively the domain of nurses and physicians, who have to solve issues and deal with situations each day as they present themselves. The social interaction that takes place on the day of surgery may imply that, despite the planning activities in the months and weeks before surgery, there is a continuous real-time adjustment process taking place in the system. This relates to a relevant topic, namely organizational structure versus governance. Provan states that network structures can be very effective in terms of learning ability, efficient resource use and problem solving capacity, but that little is known about how to control and manage these networks [42]. The fact that managing agents seem to have a relatively low integrative role in the network that was studied could be a risk. This is particularly the case because this integration lies mainly with two agents and there is little redundancy of agents performing integrative roles.

The fact that there appears to be so much social interaction also raises questions with regard to the HIS, because the IT system does not seem to replace the interaction needs of agents. This could be because IT systems, which require standardization and uniformity of operational processes [9], do not fit a reality that is much more varied and uncertain. There could also be a mismatch between the formal and informal organizational memory, in which data and knowledge are stored in both the IT system and in the heads of individuals, as stated by van Merode et al. [9]. This mentioned possible shortcoming of an IT system to present data in line with reality is in line with van Merode et al’s statement that ‘processes may fail in unpredictable ways and may be difficult to trouble-shoot and correct’ [9]. In addition, strategic decisions that impact the operational system could also have unexpected outcomes if these decisions are made without knowing how the operational network functions. We believe it is important to link the operational and the strategic systems of hospitals and study how these should be integrated and differentiated.

The findings of this case study raise the important question as to what extent the logistical hospital system generally functions as described here. A clear limitation of this study is that this is a first case, both in using a system-wide perspective as well as the application of social network analysis theory to do so. Furthermore, the exclusion of external agents and the patients in our research limits our perspective on the functioning of the system in relation to its environment. A third limitation is the fact that interaction frequency and specific time aspects were excluded; consequently, the importance of one interaction over the other is not identified. Last but not least, based on this research, we do not know how network structure relates to the hospital’s performance, which is highly relevant, since improving hospital performance is an important motivation for this type of study. However, in line with Yin, this exploratory study has seized the opportunity ‘to shed empirical light on some theoretical concepts or principles’ [28]. Rather than generalizing these findings statistically, this case study should be used for analytic generalization [28], either by defining new research or by reinterpreting other studies or cases in this field. There are several issues we propose to explore further.

First, an important question to consider is what is the relation between the network structure, in particular integration and differentiation and its performance. In social network theory, several statements have been made regarding the efficiency, effectiveness, flexibility and vulnerability of networks. Kilduff [17], for example, states that a clique ‘represents maximum inefficiency’. Although this statement was put forth in a technical treatise of network structures, the redundant and repetitive interaction between agents in Slingeland Hospital raises questions with regard to the efficient use of resources, in particular concerning the efficient use of physicians and nurses. In relation to efficiency, Provan states that cliques should not be too large nor should the number be too high [16], and Volland states that relieving medical staff from activities that are not directly patient-related could improve the quality of health care [43]. On the other hand, the high number of cliques and redundancy may be effective in dealing with the complexity of the logistics of surgery. The fact that surgeons, OR nurses and nurse anesthetists collaborate in many different cliques may increase network flexibility. In order to assess hospital performance we believe a new framework is required, which must include multiple parameters relating to the interests of individual agents and the various demands stemming from the hospital’s environment, both within parts of the hospital network as well as on a hospital-wide level [13].

A second topic is how integration is best achieved in hospitals. There are vulnerabilities in the network of Slingeland Hospital, because without the OTC capacity planner and the OTC day coordinator the system would fall into fragmented parts, creating so-called structural holes [17]. In addition, as nurses, surgeons and anesthesiologists perform integrative tasks, it may be a burden for them to perform tasks directly related to patient care. Also, it’s important to address the role of management and brokers and whether they should coincide or not. Further research is necessary to determine how many brokers are required and how they should be positioned in relation to managers, physicians and nurses.

In addition, given the statements of Lawrence and Lorsch [15] and Provan [16] that integration and differentiation should fit the demands of the hospital environment, it is important to examine how the network structure fits the demands that are put on the hospital system, not just by patients but also by policy makers, insurance companies and other stakeholders. Van Merode et al. also state that organizations, ‘according to contingency theory should adopt a mechanistic form if their task is simple and stable and their goal is efficiency and they should adopt an organic form if their task is complex and changing and their goal is therefore flexibility’ [9]. From this network analysis no clear distinction between simple or complex tasks emerged. Following van Merode et al’s statement that a different control system should be designed if there is no homogeneity in the hospital’s services [9], it is important to study further the distinction between the more ‘mechanistic’ part of the hospital and tasks requiring more ‘organic’ flexible structures.

A third research area is how social network theory and the associated metrics relate to the concepts of integration and differentiation. The metrics used here represent a mathematical value for integration and need to be linked to concepts in organization theory. The concepts used in organization theory and social network analysis are not always the same. It is important to note, for example, that “centralization” as defined by Mintzberg [40] is not the same as centralization in social network theory. Mintzberg associates centralization with decision power. For instance, “vertical centralization” entails that decision-making power is centrally located at the strategic apex of an organization. An agent who is in a central position in a social network can be a member of the strategic apex, but this is not necessarily the case. For differentiation the concepts of social network theory are even less clear. In this study we explored these concepts, but we believe this has to be studied further for developing social network theory.

The main strength of this study is that it presents a new perspective on the hospital’s logistical system and responds to the statement that most studies fail to address the entire hospital supply chain or network [1]. This study also responds to the fact that hospital-wide studies have not been performed using quantitative techniques, and optimization is often based on ‘policy’ and ‘experience’ rather than on data [43]. Although we have not studied optimization, this study could be a fruitful basis for doing that, thereby developing logistical and organizational theories that are coherent with the hospital’s practice.

## Conclusions

In conclusion, this social network analysis of a hospital’s logistical network, the first as far as we know, sets a basis for further research on integration and differentiation. It identifies a possible gap between existing organizational perspectives on hospitals and the reality. This should be analyzed further in order to be able to increase the effectiveness of hospitals. A first step would be to replicate the methods applied here in other hospitals. More case study research in the future would enable academia to develop new theories on the organization of hospitals. This knowledge is important for healthcare policy makers and for the strategic management of hospitals; it can support the effective integration and differentiation of tasks in both the operational system and the strategic system, within hospitals or even in regional healthcare alliances.

## Supplementary information

**Additional file 1.** Data collected and used for social network analysis per task. Additional file 1 shows which sources provided the input for establishing the interactions for each task.

**Additional file 2. **Social network per task. In additional file 2 each task is described in detail and the social network structure of agents and interactions that are in place to execute the task are presented. In addition related social network metrics are presented. **Figure 1.** Social network of Task 1: Making the OR master schedule. **Figure 2.** Social network of Task 2: Making the clinical bed plan. **Figure 3.** Social network of Task 3: Scheduling surgeons and anesthesiologists. **Figure 4.** Social network of Task 4: Scheduling OTC nurses. **Figure 5.** Social network of Task 5: Planning equipment maintenance. **Figure 6.** Social network of Task 6: Planning surgery. **Figure 7.** Social network of Task 7: Order materials. **Figure 8.** Social network of Task 8: Preoperative screening. **Figure 9.** Social network of Task 9: Make appointment. **Figure 10.** Social network of Task 10: Plan OTC nurses. **Figure 11.** Social network of Task 11: Control planning. **Figure 12.** Social network of Task 12: Picking materials. **Figure 13.** Social network of Task 13: Emergency admission. **Figure 14.** Social network of Task 14: Prepare patient on nursing ward. **Figure 15.** Social network of Task 15: Prepare patient in holding. **Figure 16.** Social network of Task 16: Making radiology image. **Figure 17.** Social network of Task 17: Collaborating in the OR. **Figure 18.** Social network of task 18: Cleaning the OR. **Figure 19.** Social network of task 19: Order emergency CSD services. **Figure 20.** Social network of task 20: Patient care in recovery. **Figure 21.** Social network of task 21: Aftercare of patient. **Figure 22.** Social network of task 22: Managing the OTC day program. **Figure 23.** Social network of task 23: Manage OTC task.

**Additional file 3.** Description and degree of agents in social networks. The code names, names and network degree of all agents are presented in Additional File 3.

## Data Availability

The datasets generated and/or analyzed during the current study are not publicly available for reasons of confidentiality, but are available on reasonable request from corresponding author Annelies van der Ham who can be contacted by email: a.vanderham@maastrichtuniversity.nl.

## Abbreviations

CSD: Central Sterilization Department
ENT: Ear, Nose, Throat
ER: Emergency Room
HIS: Hospital Information System
IC: Intensive Care
MIS: Medical Instrumental Services
MSC: Medical Specialty Company
OR: Operating Room
OTC: Operating Theatre Complex
