# Supplementary material for: Identifying integration and differentiation in a Hospital’s logistical system: a social network analysis of a case study
Source: BMC Health Serv Res. 2020 Sep 11;20:857. doi: 10.1186/s12913-020-05514-w (PMC7488445; doi:10.1186/s12913-020-05514-w)
Supplement: Supplementary file 1 — Additional file 1. Data collected and used for social network analysis per task. Additional file 1 shows which sources provided the input for establishing the interactions for each task. [file 12913_2020_5514_MOESM1_ESM.docx]

**Additional file 1: Data collected and used for social network analysis per task**

| **#** | **Task** | **HIS data** | **Documents** | **Observations** | **Interview** |
| --- | --- | --- | --- | --- | --- |
| 1 | Make OTC master schedule |  | - Internal presentation on working procedures - OTC master schedules |  | - OTC capacity planner - Surgeon - Anesthesiologist |
| 2 | Make clinical bed plan |  | - Clinical bed plan - Internal presentation on clinical bed planning |  | - OTC capacity planner |
| 3 | Schedule surgeons and anesthesiologists | - Number of surgeons and anesthesiologists involved in surgeries in 2017 | - Number of surgeons and anesthesiologists on hospital website | - Three outpatient departments with 7 secretaries - Preoperative department with 1 secretary | - 2 surgeons - 1 anesthesiologist - OTC capacity planner |
| 4 | Schedule OT nurses | - Number of OCT nurses involved in surgeries in 2017 | - Working schedules OTC nurses - Overview of OTC nurses - Clusters of OTC nurses - Sign up forms working shifts - Overview of working day preferences |  | - 3 team leaders of the OTC |
| 5 | Plan equipment maintenance |  |  |  | - OTC capacity planner |
| 6 | Plan surgery | - Number of surgeons and anesthesiologists involved in surgeries in 2017 | - Number of surgeons and anesthesiologists on hospital website - Planning rules for general surgery - Time out procedure | - Three outpatient departments with 6 secretaries | - OTC capacity planner - 2 surgeons - 1 anesthesiologist |
| 7 | Order materials |  | - Overview of OTC nurses responsible for ordering materials for specific surgeries | - 3 outpatient departments with 6 secretaries - OTC day coordinator | - Team leader of the OTC - OTC capacity planner |
| 8 | Preoperative screening | - Number of anesthesiologists involved in surgeries in 2017 | - Overview of staff in preoperative screening - Time out procedure | - Preoperative screening department with preoperative secretary, preoperative nurse, - pharmacy assistant and anesthesiologist | - OTC capacity planner |
| 9 | Make appointment |  | - Overview of staff in preoperative screening | - Preoperative secretary - 3 outpatient departments with 6 secretaries |  |
| 10 | Plan OT nurses |  | - Overview number of OTC nurses - Planning of OTC nurses | - OTC day coordinator | - Team leader of the OTC |
| 11 | Control planning |  | - Planning checklist OTC program | - OTC day coordinator - Surgeries in two Operating Rooms - 3 outpatient departments with 6 secretaries | - OTC capacity planner |
| 12 | Pick materials |  |  | Logistical staff on OTC |  |

| **#** | **Task** | **HIS data** | **Documents** | **Observations** | **Interview** |
| --- | --- | --- | --- | --- | --- |
| 13 | Emergency admission |  |  | OTC day coordinator | - Team leader Emergency Department |
| 14 | Prepare patient on ward | Surgery registration data including the ward for each patient | - Overview of number of ward nurses for each nursing ward - Day program - Time out procedure | 2 nursing wards |  |
| 15 | Prepare patient in holding | Surgery registration data of 2017 | - Holding checklists - Process flow scheme holding - Time out procedure | Holding |  |
| 16 | Make Radiology image |  |  | - Surgeries in two Operating Rooms Recovery |  |
| 17 | Doing surgery | - Surgeons, anesthesiologists, OTC staff involved in each surgery in 2017 | - Time out procedure | - Surgeries in two Operating Rooms - OTC day coordinator | - 2 surgeons - 1 anesthesiologist |
| 18 | Clean OR |  |  | - Surgeries in two operating rooms | - OTC capacity planner |
| 19 | Order emergency CSD services |  |  | - OTC day coordinator - Central Sterilization Department |  |
| 20 | Patient care recovery | - Surgery registration data | - Time out procedure | Recovery | - Team leader Recovery |
| 21 | Aftercare of patient | - Surgery registration data including the ward for each patient, surgeons involved in the surgery and patient transfers | - Overview of number of ward nurses for each nursing ward - Time out procedure | - 2 nursing wards | - 2 surgeons |
| 22 | Manage OT day program |  |  | - OTC day coordinator - Surgeries in two Operating Rooms | - 1 anesthesiologist - 2 surgeons |
| 23 | Manage OT tasks |  | - Emails including planning of management meetings |  | - Cluster manager OTC and Services - OTC capacity planner - 3 team leaders of the OTC |
