# Supplementary material for: Identifying integration and differentiation in a Hospital’s logistical system: a social network analysis of a case study
Source: BMC Health Serv Res. 2020 Sep 11;20:857. doi: 10.1186/s12913-020-05514-w (PMC7488445; doi:10.1186/s12913-020-05514-w)
Supplement: Supplementary file 2 — Additional file 2. Social network per task. In additional file 2 each task is described in detail and the social network structure of agents and interactions that are in place to execute the task are presented. In addition related social network metrics are presented. Figure 1. Social network of Task 1: Making the OR master schedule. Figure 2. Social network of Task 2: Making the clinical bed plan. Figure 3. Social network of Task 3: Scheduling surgeons and anesthesiologists. Figure 4. Social network of Task 4: Scheduling OTC nurses. Figure 5. Social network of Task 5: Planning equipment maintenance. Figure 6. Social network of Task 6: Planning surgery. Figure 7. Social network of Task 7: Order materials. Figure 8. Social network of Task 8: Preoperative screening. Figure 9. Social network of Task 9: Make appointment. Figure 10. Social network of Task 10: Plan OTC nurses. Figure 11. Social network of Task 11: Control planning. Figure 12. Social network of Task 12: Picking materials. Figure 13. Social network of Task 13: Emergency admission. Figure 14. Social network of Task 14: Prepare patient on nursing ward. Figure 15. Social network of Task 15: Prepare patient in holding. Figure 16. Social network of Task 16: Making radiology image. Figure 17. Social network of Task 17: Collaborating in the OR. Figure 18. Social network of task 18: Cleaning the OR. Figure 19. Social network of task 19: Order emergency CSD services. Figure 20. Social network of task 20: Patient care in recovery. Figure 21. Social network of task 21: Aftercare of patient. Figure 22. Social network of task 22: Managing the OTC day program. Figure 23. Social network of task 23: Manage OTC task. [file 12913_2020_5514_MOESM2_ESM.docx]

**Additional file 2: Social network per task**

1. **Make the OR master schedule**


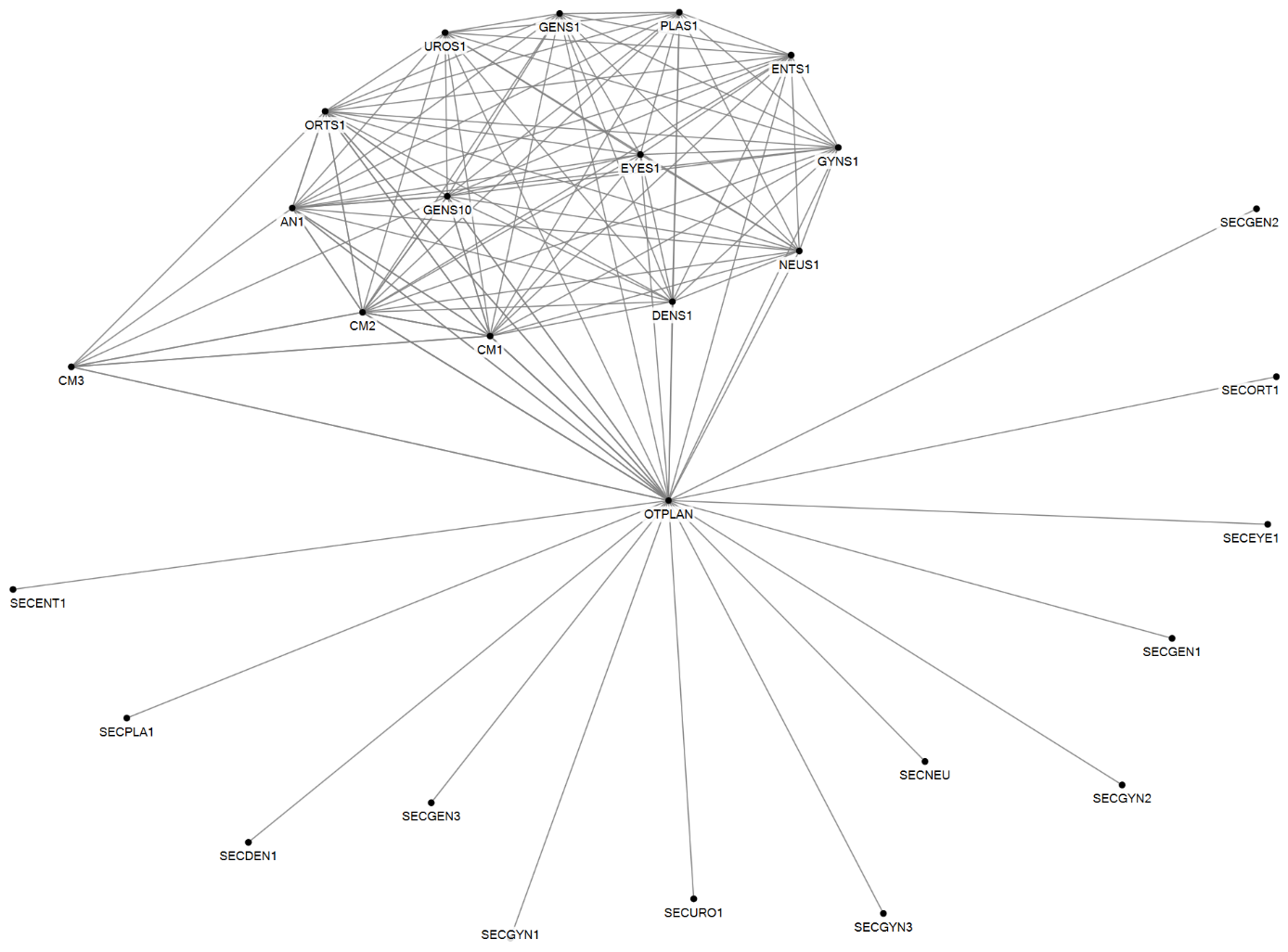


**Figure 1** Social network of Task 1: Making the OR master schedule

| **Parameter** | **Value** |
| --- | --- |
| Number of agents | 28 |
| Number of unique ties | 110 |
| Density | 0.29 |
| Number of cliques | 2 |
| Highest betweenness centrality | OTPLAN |

The Operating Room (OR) master schedule is made in the Tactical Planning Meeting (TPM). Three cluster managers who are responsible, respectively, for outpatient, inpatient departments and Operating Theatre Complex (OTC), three general surgeons, an orthopedic surgeon, an anesthesiologist and the OTC capacity planner participate in this meeting. The OR master schedule is prepared by four participants of the TPM, who then propose the scheme to the entire TPM. The OR master schedule is then presented to the OTC commission. The OTC commission discusses and advises OTC management on planning, staff and budget issues. The OTC commission includes one surgeon from every surgical discipline, the cluster manager responsible for the OTC and the OTC capacity planner. When the session schedule is final, the OTC capacity planner informs all outpatient secretaries of all changes made.

1. **Make the clinical bed plan**


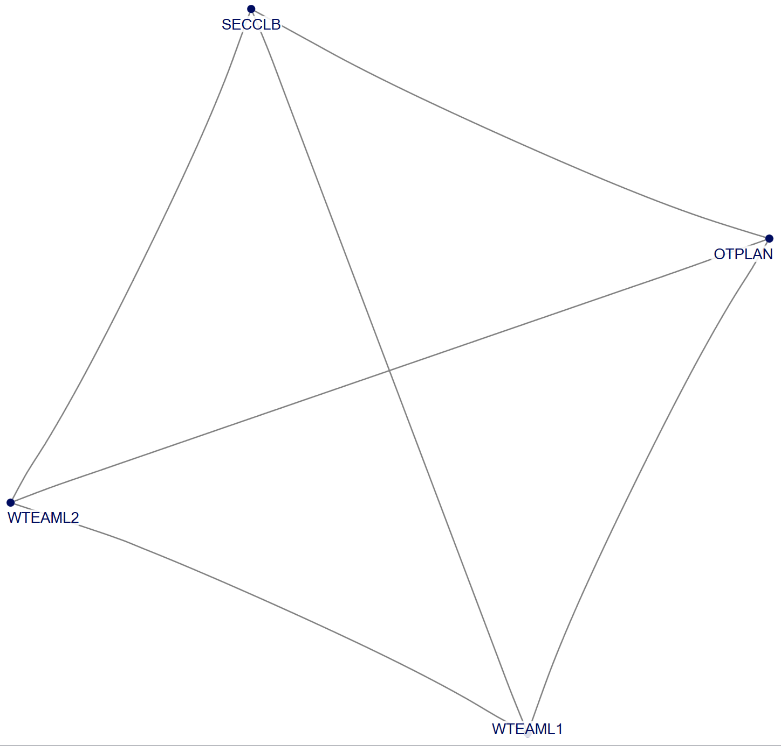


**Figure 2** Social network of Task 2: Making the clinical bed plan

| **Parameter** | **Value** |
| --- | --- |
| Number of agents | 4 |
| Number of unique ties | 6 |
| Density | 1 |
| Number of cliques | 1 |
| Highest betweenness centrality | N/A |

In the clinical bed plan, beds are assigned to a medical discipline for each nursing department. The clinical bed plan is established in consultation between the OTC capacity planner, one secretary from the nursing department and the two team leaders of the three nursing wards who host most surgery patients. The team leaders of these nursing wards are also involved in making the clinical bed plan, but one nurse ward secretary has the informal role of ‘clinical bed plan boss’. She uses this plan to correct outpatient clinics when they take a bed they cannot claim.

1. **Schedule surgeons and anesthesiologists**


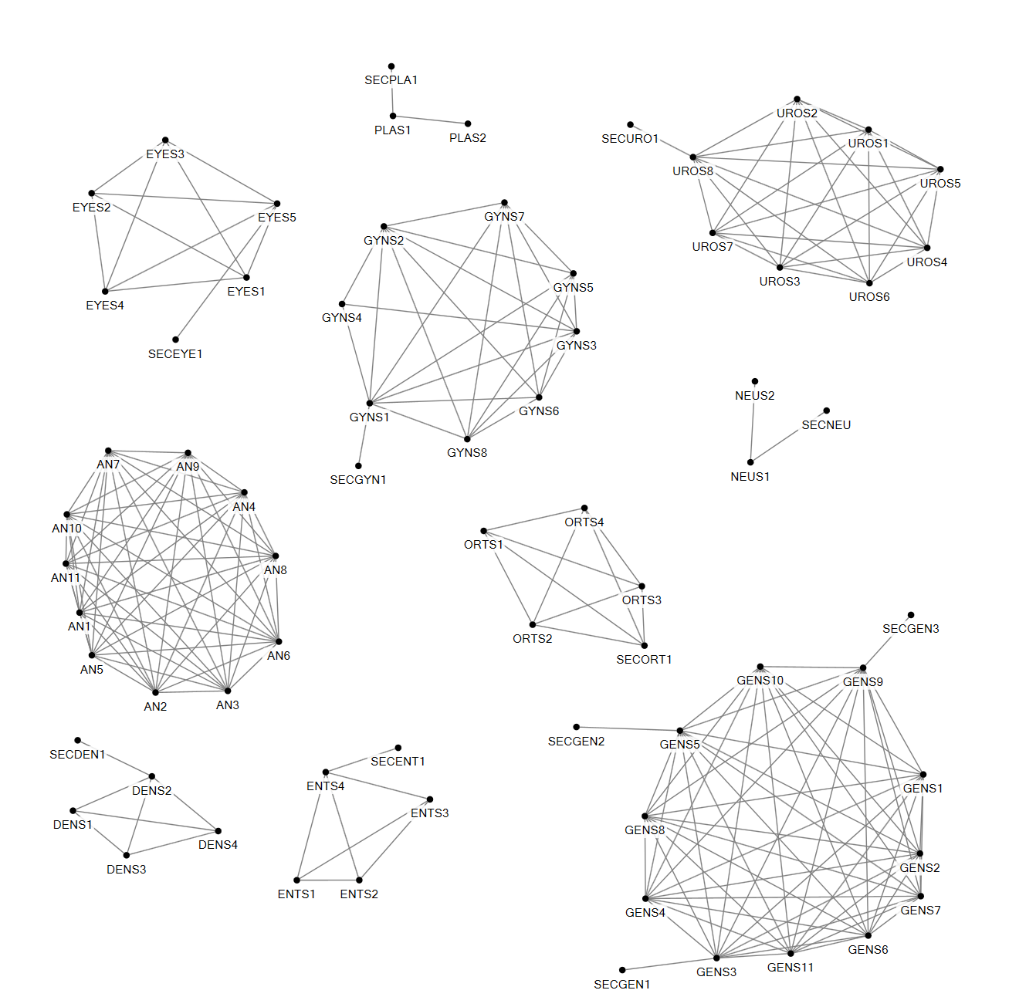


**Figure 3** Social network of Task 3: Scheduling surgeons and anesthesiologists

| **Parameter** | **Value** |
| --- | --- |
| Number of agents | 70 |
| Number of unique ties | 206 |
| Density | 0.09 |
| Number of cliques | 10 |
| Highest betweenness centrality | GENS3/GENS9 |

The OR master schedule is also used for preparing staff planning schemes. The surgeons of each outpatient department and anesthesiologists make these schedules for themselves and allocate surgeons to the time slots in the OR master schedule. One surgeon within each medical discipline group proposes the schedule and discusses it with the other surgeons. In some outpatient departments the secretary of the outpatient department is involved in this.

1. **Schedule OTC nurses**


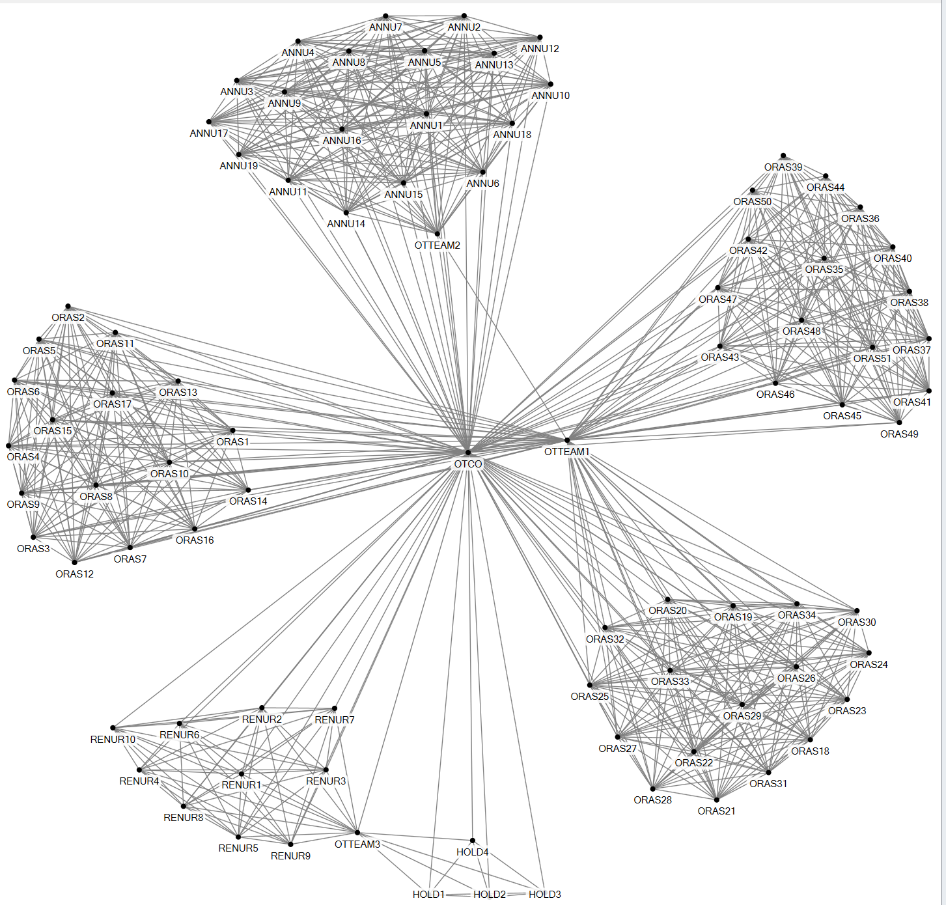


**Figure 4** Social network of Task 4: Scheduling OTC nurses

| **Parameter** | **Value** |
| --- | --- |
| Number of agents | 88 |
| Number of unique ties | 801 |
| Density | 0.2 |
| Number of cliques | 6 |
| Highest betweenness centrality | OTCO |

The OTC surgery team leader plans the schedule with the OR nurses. He makes a schedule based on the OR master schedule and communicates with OR nurses about it. In this planning process a number of factors which require interaction are taken into account. OR nurses sign up for shifts outside regular operating times on nights and weekends. The surgery team leader verifies if the nurses have signed up for the agreed number of shifts and, if necessary, makes changes in this scheme to create a fair division of shifts. When all shifts are filled, the team leader fills in the OR day schedule, taking into account the clusters of medical disciplines to which each OR nurse belongs. He also takes into account the employees who want to work on fixed days and after that the shifts for the more flexible nurses are planned. In the event of an expected shortage of OR nurses, the team leader sends an email to the OR nurses, asking them to volunteer for that particular day. If they do not volunteer, the surgery team leader picks someone. The complete OR schedule is then released to all OR nurses via the IT system. At this point OR nurses are allowed to exchange hours among themselves within the three clusters of medical disciplines. If a 'deal' has been concluded between two or more OR assistants and the date at issue is at least two weeks in advance, they make a proposal to the surgery team leader, who assesses this and agrees. If it concerns a date within the next two weeks, the proposal is presented to the OTC day coordinator and he approves or disapproves it. The OTC day coordinator is responsible for the daily operation of the OTC, making sure that all surgeries are performed according to plan.

The OTC anesthesia team leader makes the schedule for the nurse anesthetists and the team leader holding and recovery makes the schedule for the holding and recovery nurses following a similar procedure.

1. **Plan equipment maintenance**


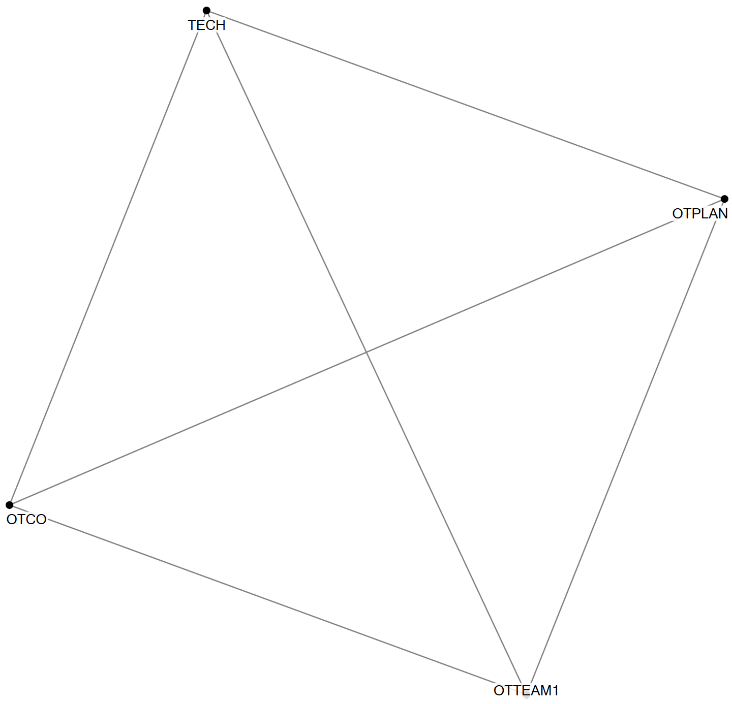


**Figure 5** Social network of Task 5: Planning equipment maintenance

| **Parameter** | **Value** |
| --- | --- |
| Number of agents | 4 |
| Number of unique ties | 6 |
| Density | 1 |
| Number of cliques | 1 |
| Highest betweenness centrality | none |

Planning is also made for OTC equipment maintenance. Technical staff from the Medical Equipment Department send an email to the OTC capacity planner with a request to perform maintenance on specific equipment. The OTC capacity planner checks with the OTC day coordinator and the surgery team leader whether this is possible, given the OR master schedule and (expected) surgeries in that period of time.

1. **Plan patient**


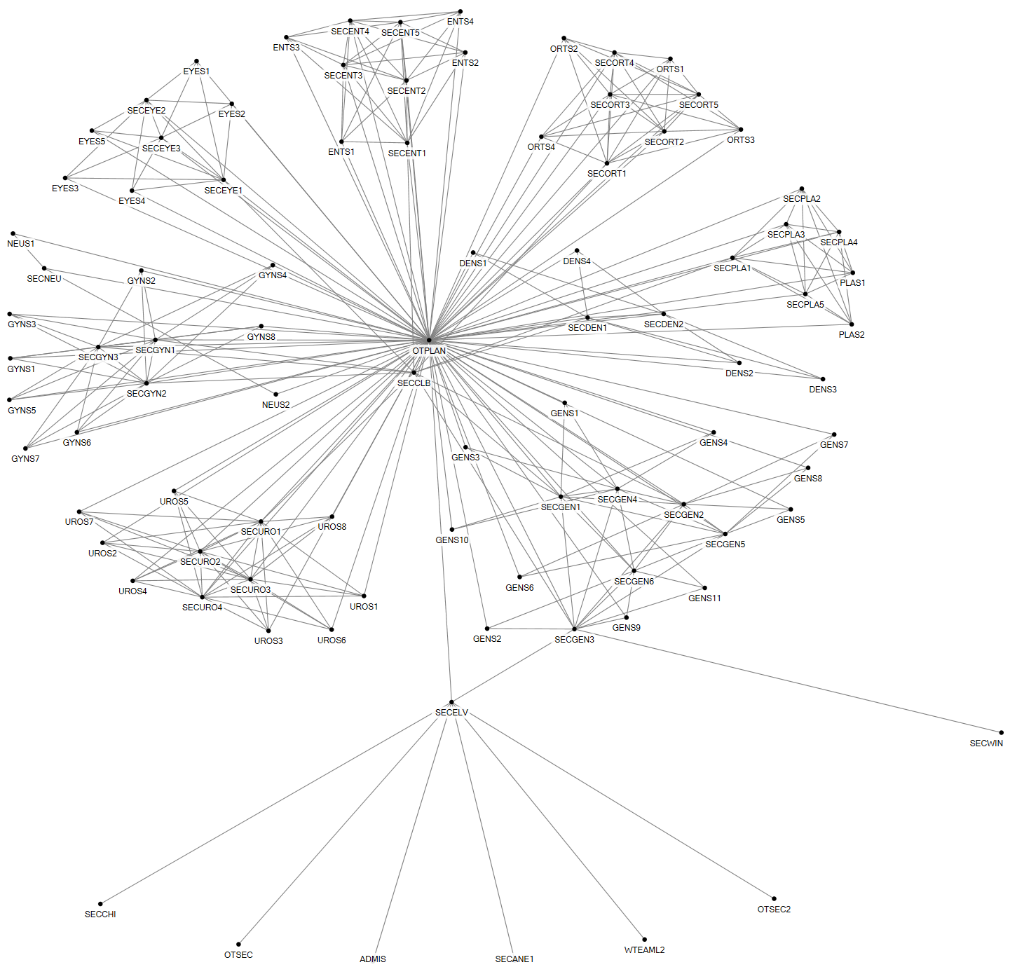


**Figure 6** Social network of Task 6: Planning surgery

| **Parameter** | **Value** |
| --- | --- |
| Number of agents | 92 |
| Number of unique ties | 315 |
| Density | 0.08 |
| Number of cliques | 48 |
| Highest betweenness centrality | OTPLAN |

The planning for surgeries takes place at one of the nine outpatient clinics after the diagnosis has been made by the physician. The surgeon agrees with the patient that he or she will be operated in an outpatient visit and takes the patient to a secretary of the outpatient department, who informs the patient about the surgery, what is to be expected and when and how the patient will be called to set a final surgery date. The secretary puts the patient on the waiting list or a surgery date right away. There can be interaction between secretaries and the OTC capacity planner on specific surgery requirements or in case the OR master schedule is almost filled. Also sessions are ‘traded’ between the secretaries and the OTC capacity planner in case sessions are under or over utilized. Surgeons also email or phone the OTC capacity planner for specific patient cases that require tuning.

Vascular patients of the Queen Beatrix Hospital are operated on in Slingeland Hospital. For these patients the outpatient secretary of the vascular surgery in the Queen Beatrix Hospital emails the vascular surgery outpatient secretary of Slingeland Hospital, who then plans these patients in.

The ‘clinical bed plan boss’ checks how the secretaries fill in the OR master schedule and how many beds are planned. If the secretaries of the outpatient departments take too many beds, she corrects them. The OTC capacity coordinator and ‘clinical bed plan boss’ evaluate how the clinical bed plan is followed and provide feedback to the secretaries on their planning activities.

1. **Request and order materials**


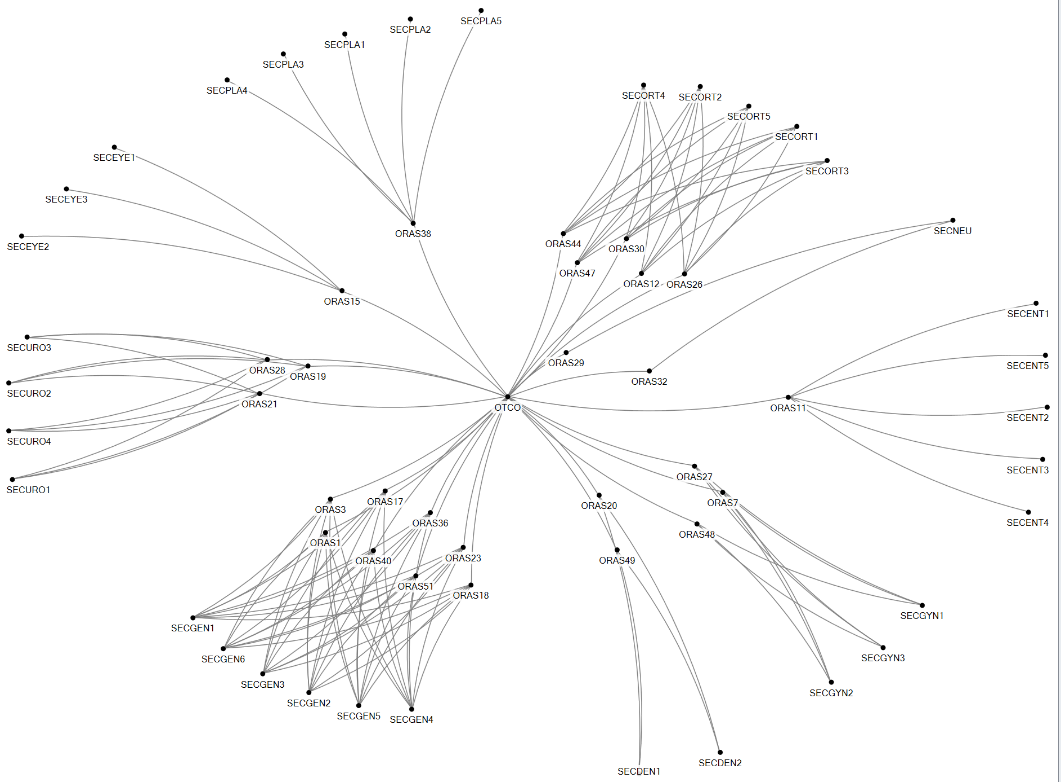


**Figure 7** Social network of Task 7: Order materials

| **Parameter** | **Value** |
| --- | --- |
| Number of agents | 61 |
| Number of unique ties | 139 |
| Density | 0.08 |
| Number of cliques | 0 |
| Highest betweenness centrality | OTCO |

If specific materials are required for an operation, the secretary of the outpatient clinic informs the OR assistant, who is specialized in the medical discipline it concerns. The OR nurse then orders the required materials via the OTC day coordinator, who then orders the materials from external suppliers via the Purchasing Department.

Other material related tasks are the cleaning of medical instruments which are delivered to the OTC by the Central Sterilization Department (CSD) every day. Further, medication, consumables and implants are delivered every day by a variety of external suppliers. One logistical staff member receives these materials and puts these in the right storage room.

1. **Preoperative screening**


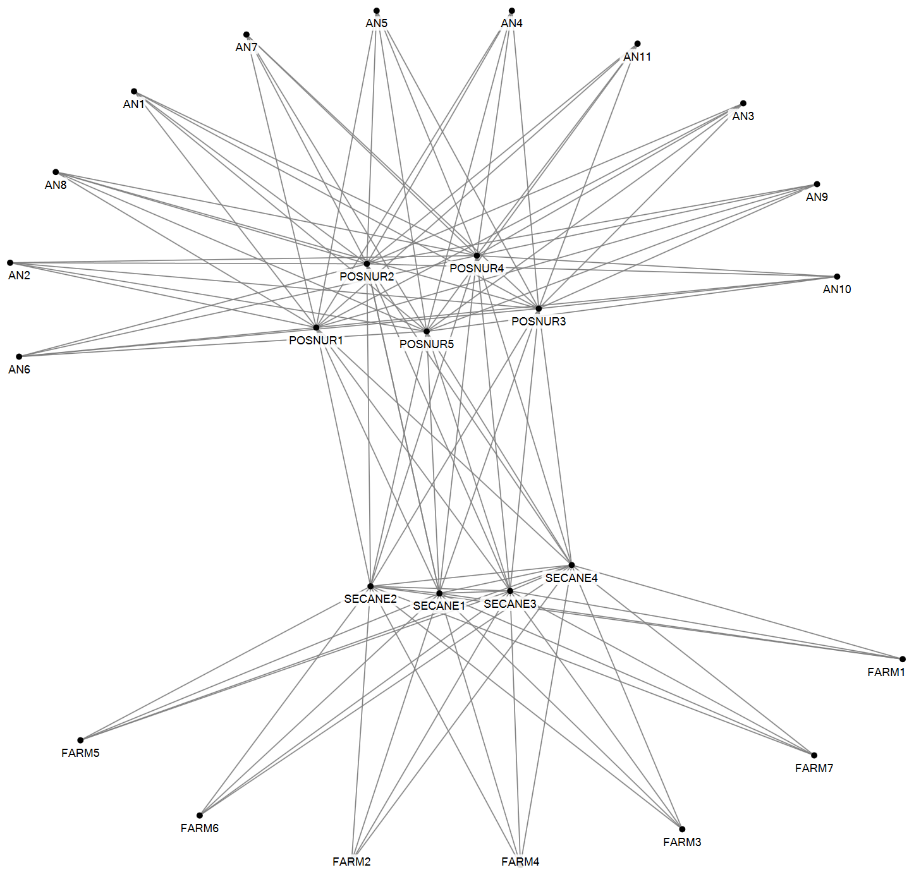


**Figure 8** Social network of Task 8: Preoperative screening

| **Parameter** | **Value** |
| --- | --- |
| Number of agents | 27 |
| Number of unique ties | 109 |
| Density | 0.31 |
| Number of cliques | 12 |
| Highest betweenness centrality | All SECAN/ POSNUR |

Before a final date can be set for the surgery, the patient must be screened by the anesthesiologist and prepared accordingly in the preoperative department. In the preoperative visit the patient first visits the pharmacy assistant (FARM), who checks which medication the patient is using. Subsequently, a physical examination is performed, after which the patient discusses with the anesthesiologist (AN) which anesthetic technique will be used, which medication he or she may continue to use on the day of the operation and then the anesthesiologist approves the operation. Right after this the patient meets the preoperative nurse (POSNUR), who further prepares the patient for surgery by providing information and discussing the preparations and aftercare the patient requires.

1. **Request and make appointment**


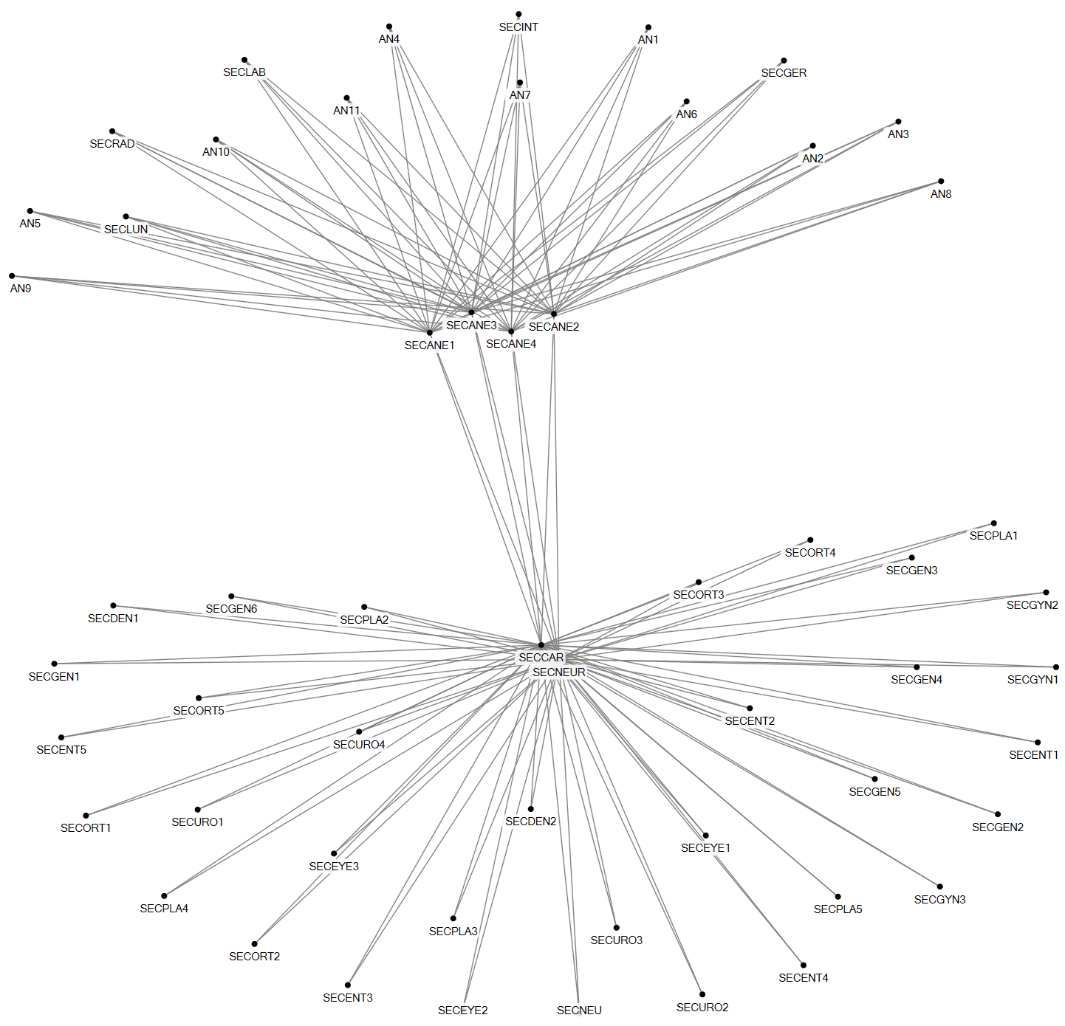


**Figure 9** Social network of Task 9: Make appointment

| **Parameter** | **Value** |
| --- | --- |
| Number of agents | 56 |
| Number of unique ties | 140 |
| Density | 0.09 |
| Number of cliques | 0 |
| Highest betweenness centrality | SECNEUR/SECCAR |

The anesthesiologist can decide, based on the patient's health situation, that the patient has to visit other physicians, prior to the operation, such as the cardiologist or the neurologist. The secretary at the pre-operative outpatient clinic arranges this for the patient, as well as any necessary blood tests or making an ECG image. Any appointments that need to be made for this are made by the secretary of the preoperative outpatient department.

After the consultation with the anesthesiologist, the patient goes to a nurse who informs the patient about the operation and prepares him or her more extensively.

In case a patient uses anticoagulants, the outpatient department of the surgeon who will perform the surgery arranges a visit to the neurologist or cardiologist.

1. **Plan OTC nurses**


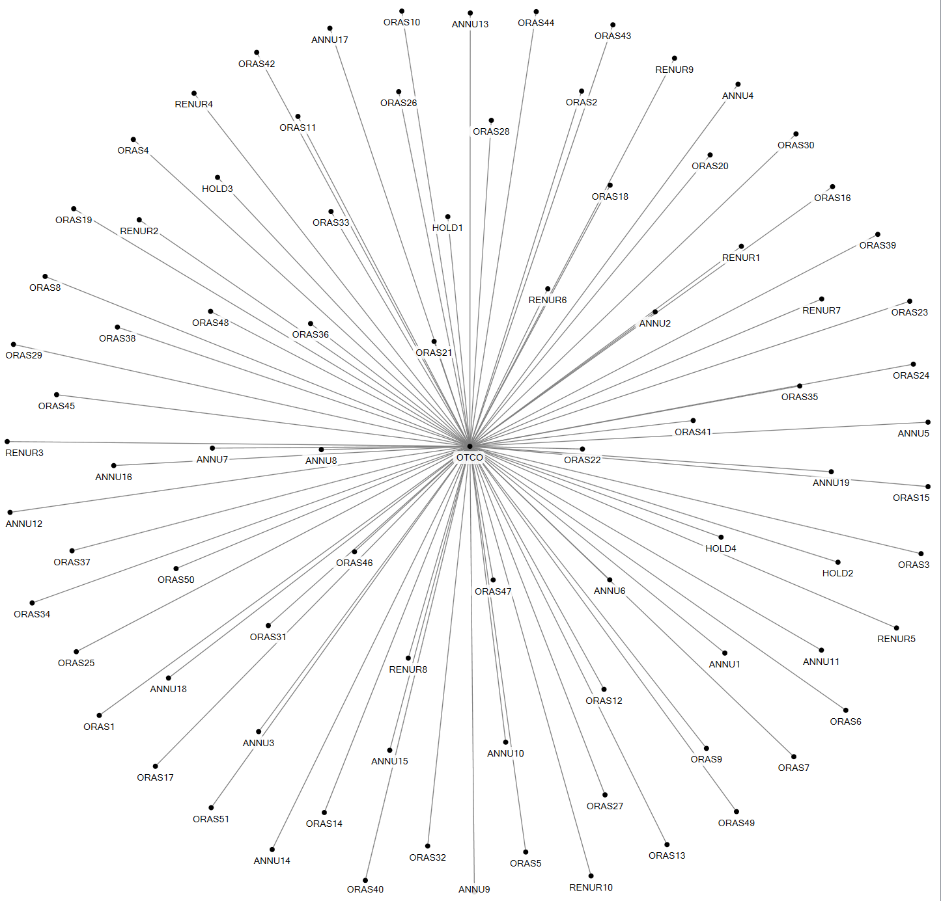


**Figure 10** Social network of Task 10: Plan OTC nurses

| **Parameter** | **Value** |
| --- | --- |
| Number of agents | 85 |
| Number of unique ties | 84 |
| Density | 0.02 |
| Number of cliques | 0 |
| Highest betweenness centrality | OTCO |

A week before surgeries are performed, the day coordinator allocates the OR nurses to surgeries for the upcoming week and he handles all communication with regard to their availability, illness or other casualties.

1. **Control planning**


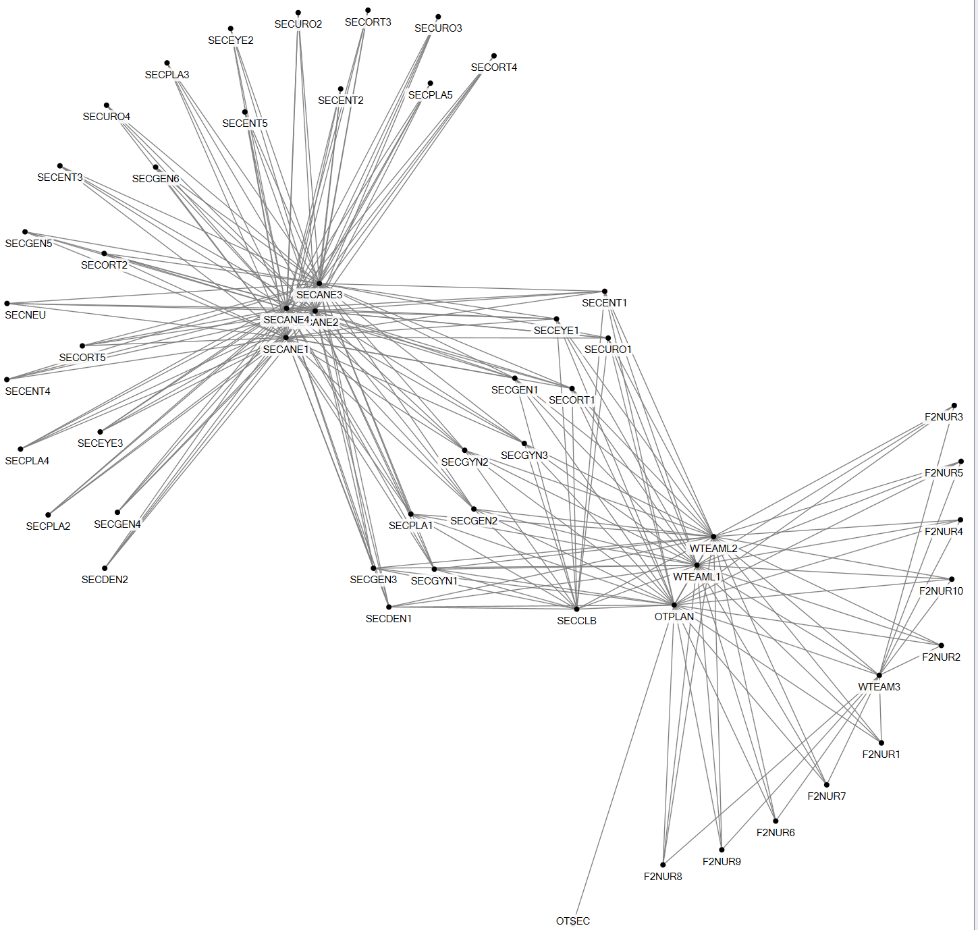


**Figure 11** Social network of Task 11: Control planning

| **Parameter** | **Value** |
| --- | --- |
| Number of agents | 54 |
| Number of unique ties | 234 |
| Density | 0.16 |
| Number of cliques | 2 |
| Highest betweenness centrality | All SECANE |

In the process towards the surgery taking place, the planning is checked and revised. Preoperative screening needs to be performed before the date of surgery is final. The preoperative secretaries interact with all outpatient secretaries on whether everything is arranged for the surgery to take place.

In the weekly ‘Tuesday morning’ meeting the planning for the upcoming week is discussed between the outpatient secretaries, the ward team leaders and the OTC capacity planner. Also a weekly bed meeting takes place between ward team leaders, a nurse and the OTC capacity planner. In this meeting all checks for the next day’s OR program are made.

The OTC capacity planner views the daily OTC schedule the day before to determine the exact sequence of the operations. For planning surgeries and determining the sequence of the procedures, a large number of control rules are set and these need to be checked. The OTC capacity planner reviews the electronic patient file of each individual patient, checking if all information has been taken into account. When everything is checked, she informs the OTC day coordinator regarding any specific details in the next day’s OTC program.

1. **Pick materials**


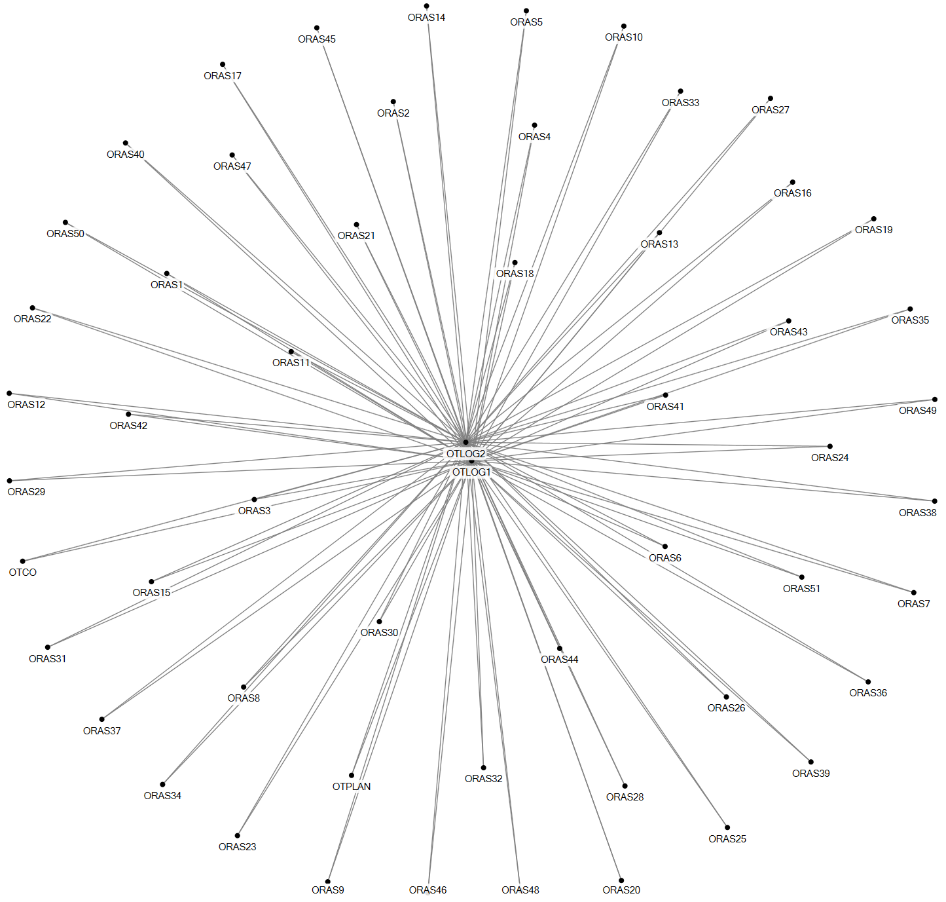


**Figure 12** Social network of Task 12: Picking materials

| **Parameter** | **Value** |
| --- | --- |
| Number of agents | 55 |
| Number of unique ties | 107 |
| Density | 0.07 |
| Number of cliques | 53 |
| Highest betweenness centrality | All OTLOG |

Medical instruments, consumables and implants are collected a day in advance by two logistical OTC staff members. Sterile and non-sterile materials stored in three storage rooms at the OTC are picked and placed in carts, which are then put in the preparation rooms which are located beside each OR. Materials are mainly collected on the basis of bills of material, which are available for every surgery. Logistical staff members consult the OTC capacity planner, OTC day coordinator and OR nurses on what particular surgeries involve, in case the bill of material doesn’t provide enough information or in case they are not familiar with a surgery type.

1. **Emergency admission**


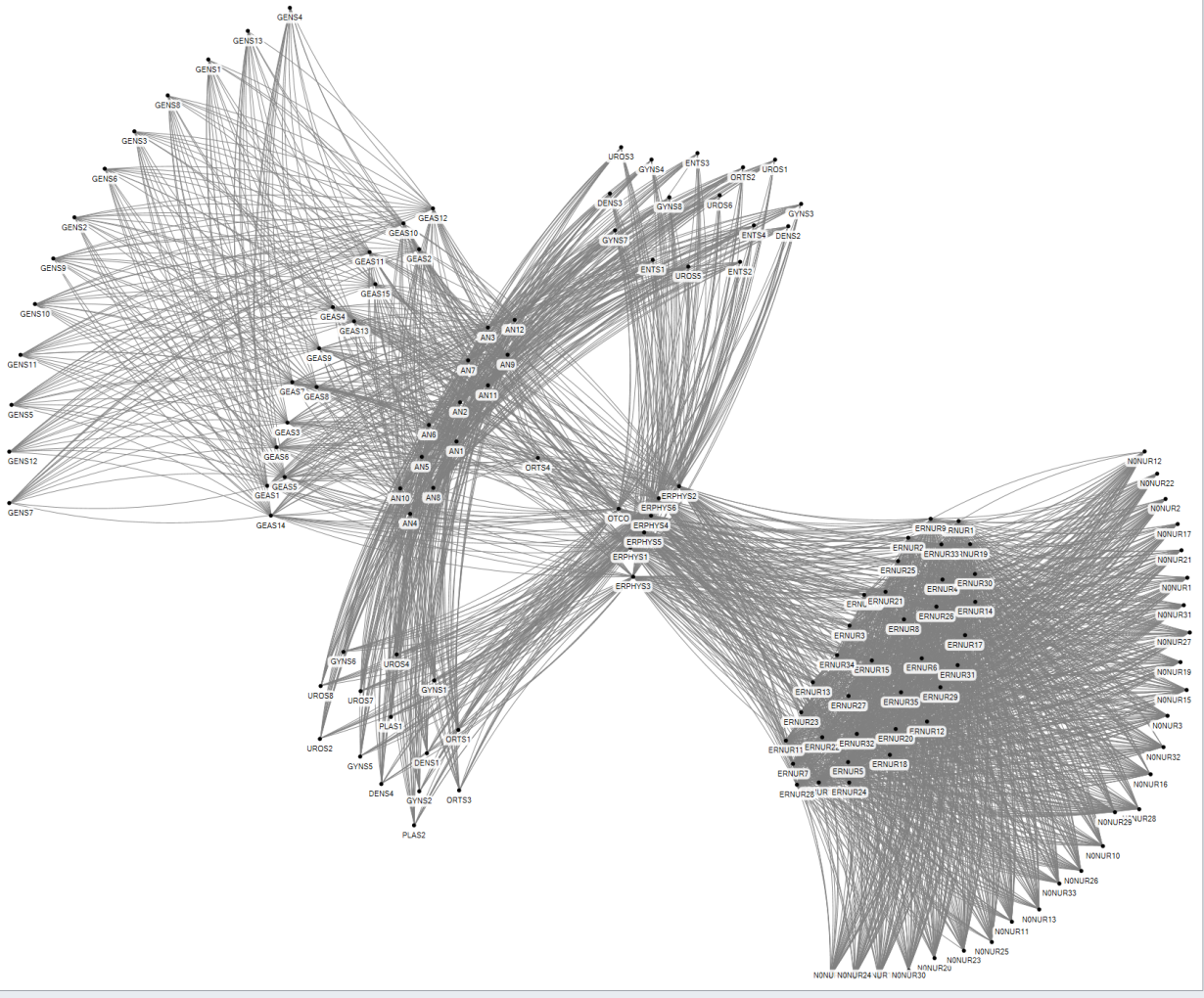


**Figure 13** Social network of Task 13: Emergency admission

| **Parameter** | **Value** |
| --- | --- |
| Number of agents | 139 |
| Number of ties | 2840 |
| Density | 0.30 |
| Number of cliques | 1 |
| Highest betweenness centrality | OTC day coordinator (OTCO) |

Patients who arrive at the Emergency Department (ED) are examined by the ED physician (ERPHYS). He calls the assistant surgeon or a surgeon if he thinks that the patient requires surgery. The assistant surgeon or surgeon comes to the ED and sets the diagnosis. If necessary blood samples or images are made. If they decide to operate, the general surgeon is called, as well as the OTC day coordinator. The ED nurse takes care of the patient and calls the OTC day coordinator as well to see if the patient goes straight to the OTC or to the nursing ward that admits emergency patients. The surgeon orders preoperative screening with the anesthesiologist, who executes this by screening the patient file. Planning the surgery into the OR program is done through communication between the OTC day coordinator, surgeon and anesthesiologist.

1. **Prepare patient on ward**


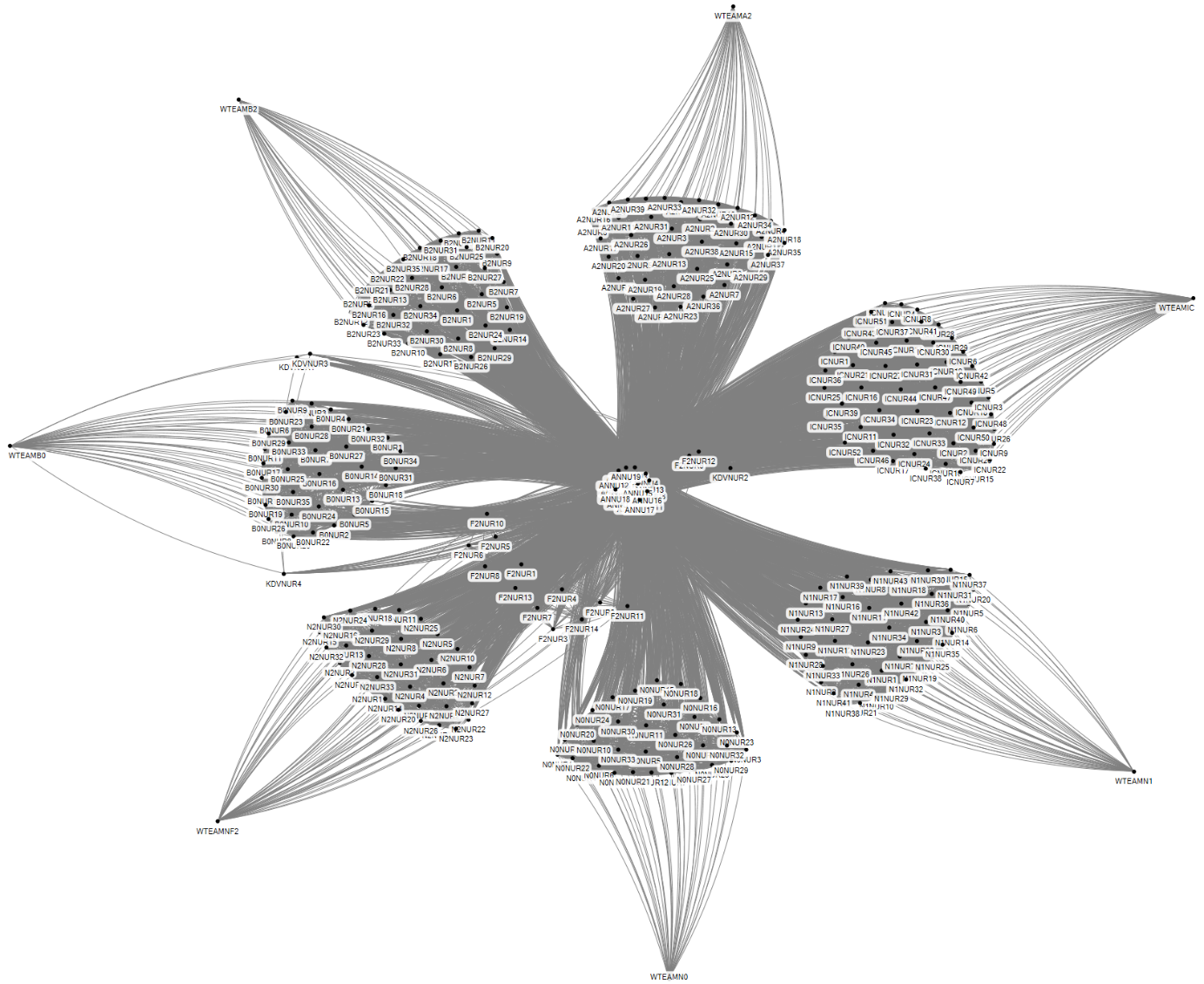


**Figure 14** Social network of Task 14: Prepare patient on nursing ward

| **Parameter** | **Value** |
| --- | --- |
| Number of agents | 314 |
| Number of ties | 11,071 |
| Density | 0.23 |
| Number of cliques | 9 |
| Highest betweenness centrality | All ANNU |

From the moment the patient is admitted to the hospital, a series of tasks including a lot of communication is performed within a short period of time. First the patient is admitted to one of the nursing wards. During the intake interview a number of checks are made to see if the patient is well prepared for the surgery. The nurse anesthetist then calls the nurse to indicate that premedication should be given to the patient, mostly 2 hours before the expected starting time of the surgery. The nurse anesthetist makes a second call to the nurse to say that the patient is to be taken to holding.

1. **Prepare patient on holding**


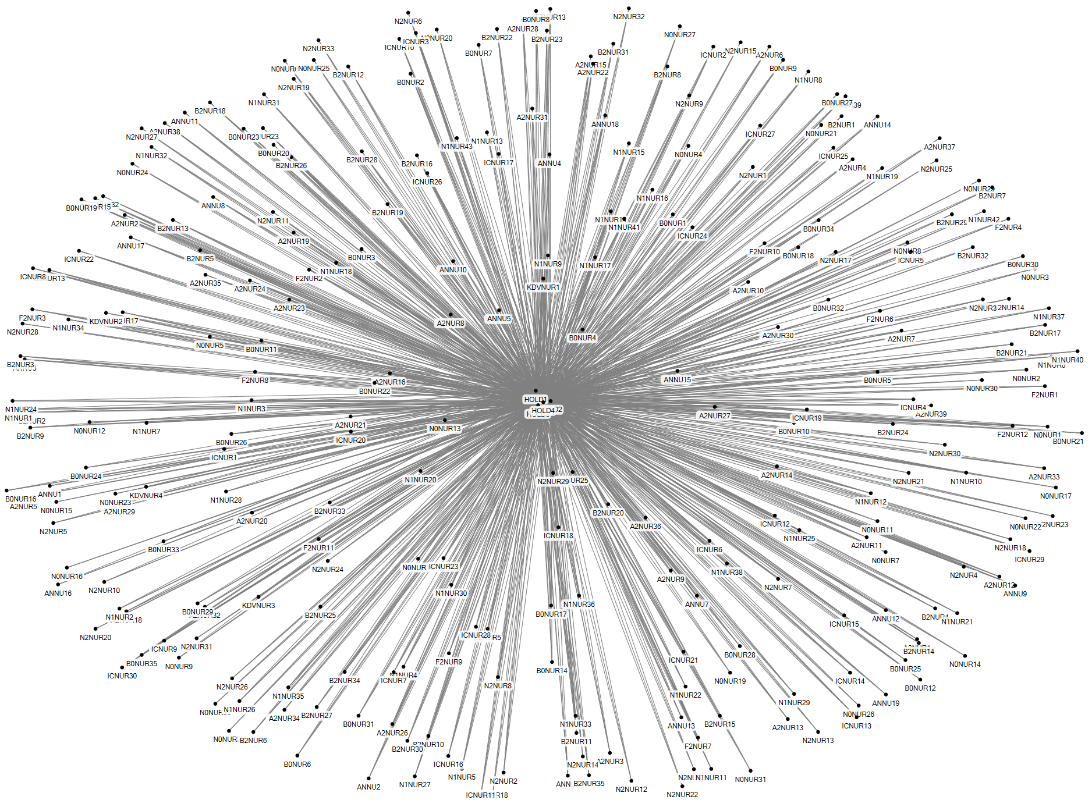


**Figure 15** Social network of Task 15: Prepare patient in holding

| **Parameter** | **Value** |
| --- | --- |
| Number of agents | 289 |
| Number of unique ties | 1100 |
| Density | 0.03 |
| Number of cliques | 65 |
| Highest betweenness centrality | All ANNU |

After the nurse hands over the patient to one of the holding nurses, following a standard transfer protocol, the second stage starts. The holding nurse prepares the patient by, among other things, preparing the infusion devices. When it is time to go to the OR the nurse anesthetist enters holding and has a small chat with the patient. The holding nurse hands the patient over to the nurse anesthetist, using the standard transfer protocol.

1. **Make Radiology image**


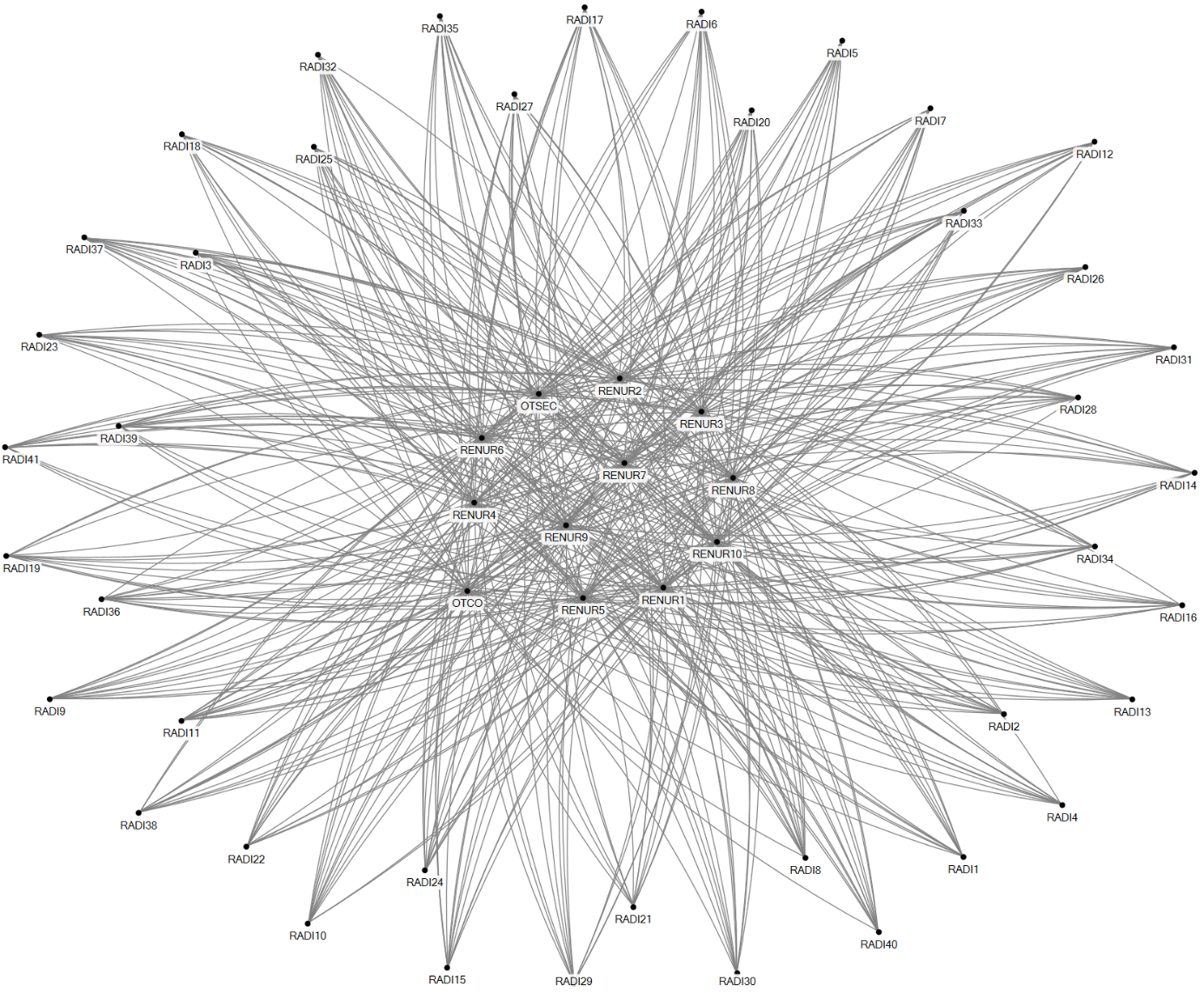


**Figure 16** Social network of Task 16: Making radiology image

| **Parameter** | **Value** |
| --- | --- |
| Number of agents | 53 |
| Number of unique ties | 491 |
| Density | 0.36 |
| Number of cliques | 0 |
| Highest betweenness centrality | All Recovery nurses, OTC day coordinator and OTC secretary |

Images can be made before or after surgery. When images are needed before surgery, the OTC day coordinator puts in a request to the radiology department, and they send someone in. When an image is required post-surgery, while the patient is in recovery, the recovery nurse calls the radiology department.

1. **Perform surgery**


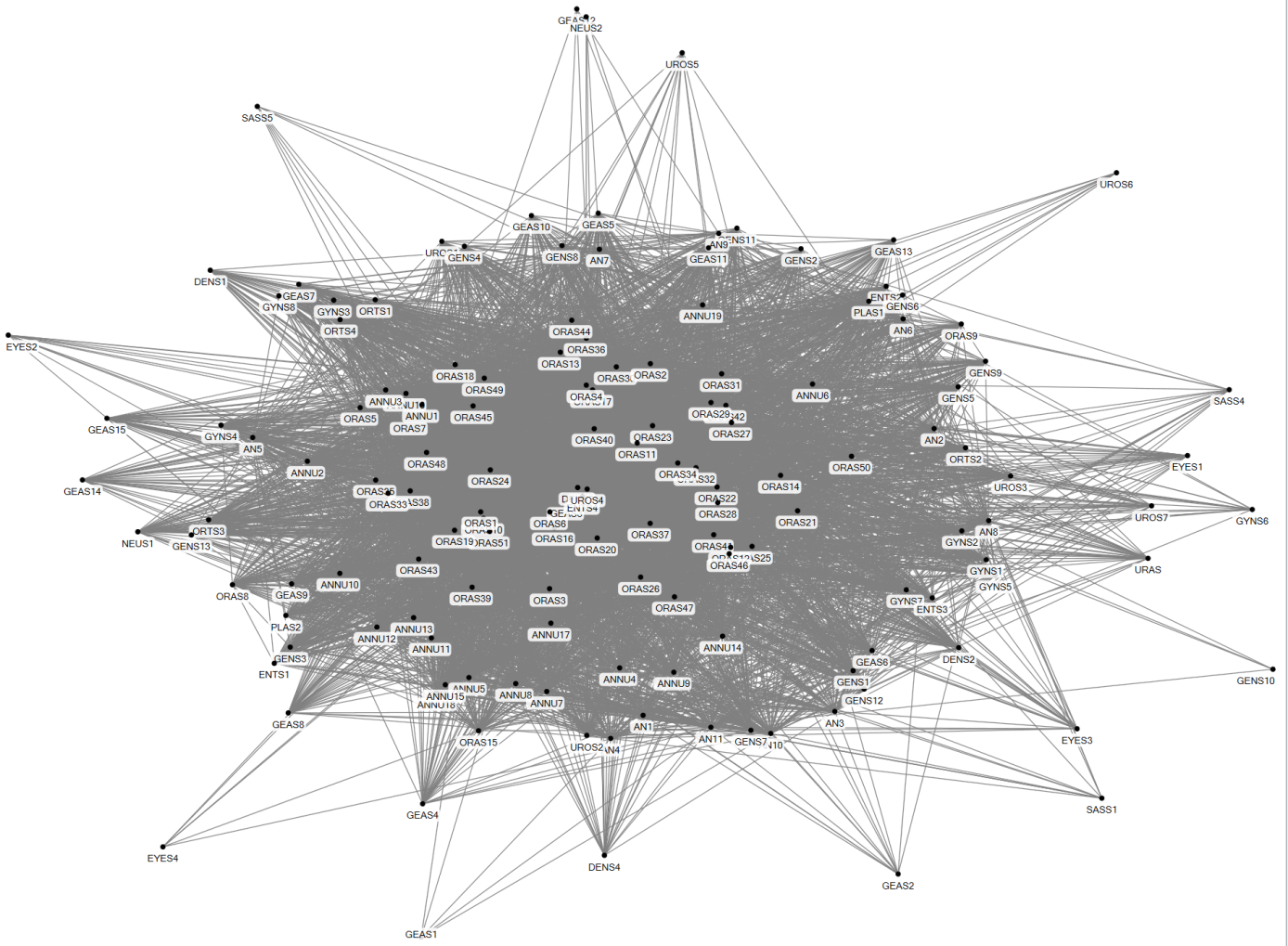


**Figure 17** Social network of Task 17: Collaborating in the OR

| **Parameter** | **Value** |
| --- | --- |
| Number of agents | 148 |
| Number of unique ties | 5444 |
| Density | 0.5 |
| Number of cliques | 7640 |
| Highest betweenness centrality | ORAS17 |

Once the patient is in the OR the nurse anesthetist mentions any relevant details about the patient to the rest of the OR team. The anesthesiologist administers anesthetics prior to the surgery and leaves once the patient is asleep. The surgery is performed by the surgeon, assisted by the OR nurses. The nurse anesthetist monitors the patient and calls the anesthesiologist if necessary.

1. **Clean the OR**


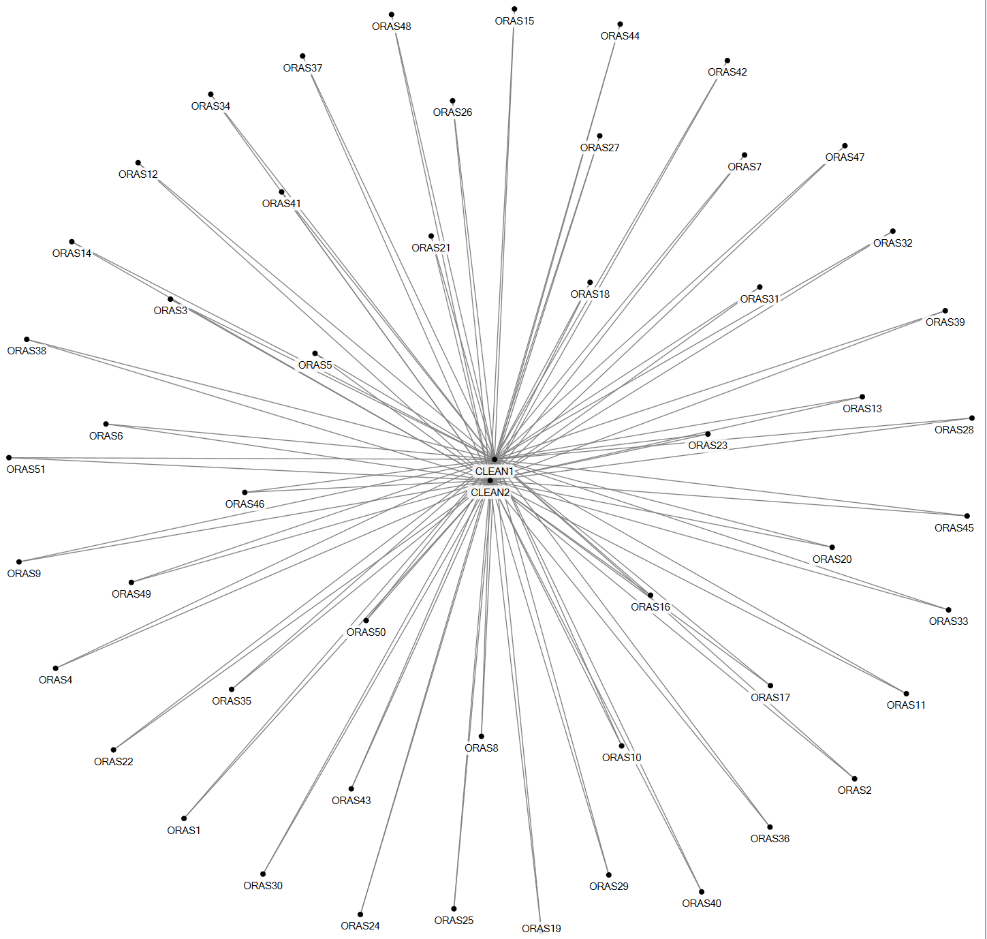


**Figure 18** Social network of task 18: Cleaning the OR

| **Parameter** | **Value** |
| --- | --- |
| Number of agents | 53 |
| Number of unique ties | 102 |
| Density | 0.07 |
| Number of cliques | 0 |
| Highest betweenness centrality | All CLEAN |

At the end of the surgery the anesthesiologist wakes the patient and the OR assistant calls the cleaning staff. If body tissue has been removed from the patient, it will be sent to pathology to investigate abnormalities.

1. **Order emergency CSD services**


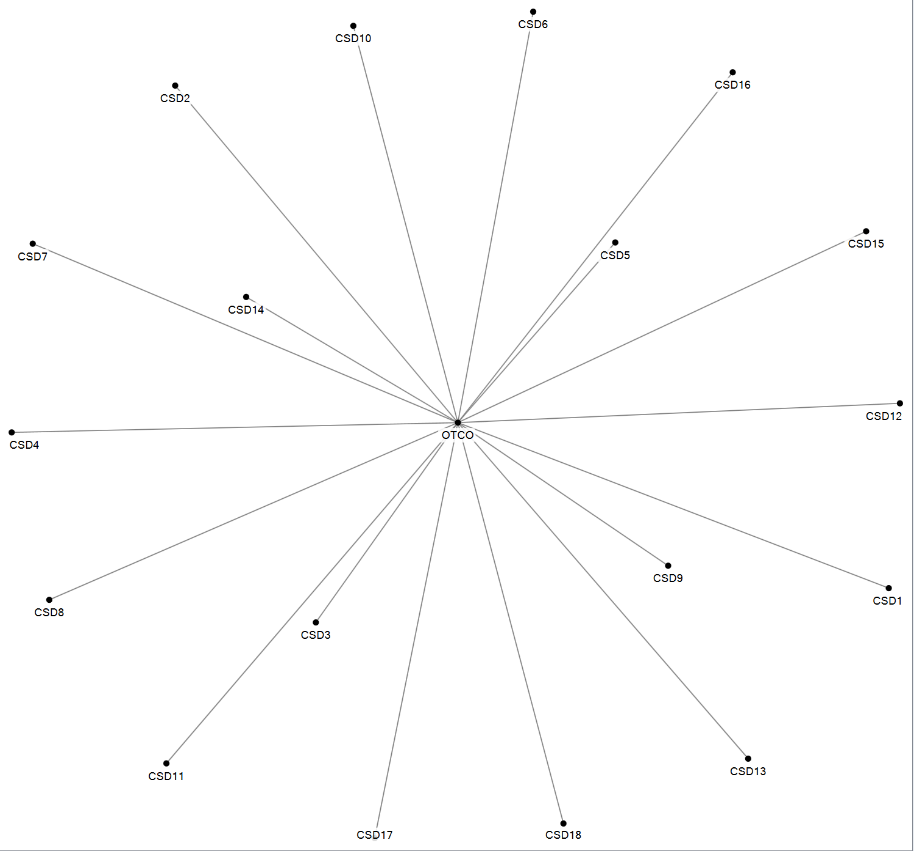


**Figure 19** Social network of task 19: Order emergency CSD services

| **Parameter** | **Value** |
| --- | --- |
| Number of agents | 19 |
| Number of unique ties | 18 |
| Density | 0.11 |
| Number of cliques | 0 |
| Highest betweenness centrality | OTCO |

After surgery an OR nurse presses a button that switches on a light at the CSD, signaling that the used medical instruments need to be collected and brought to the CSD. There is no social interaction for this, so it is not visualized in this figure. If medical instruments need to be cleaned and sterilized immediately, so they can be reused for a surgery on the same day, the OTC day coordinator calls the CSD with an emergency order.

1. **Patient care in recovery**


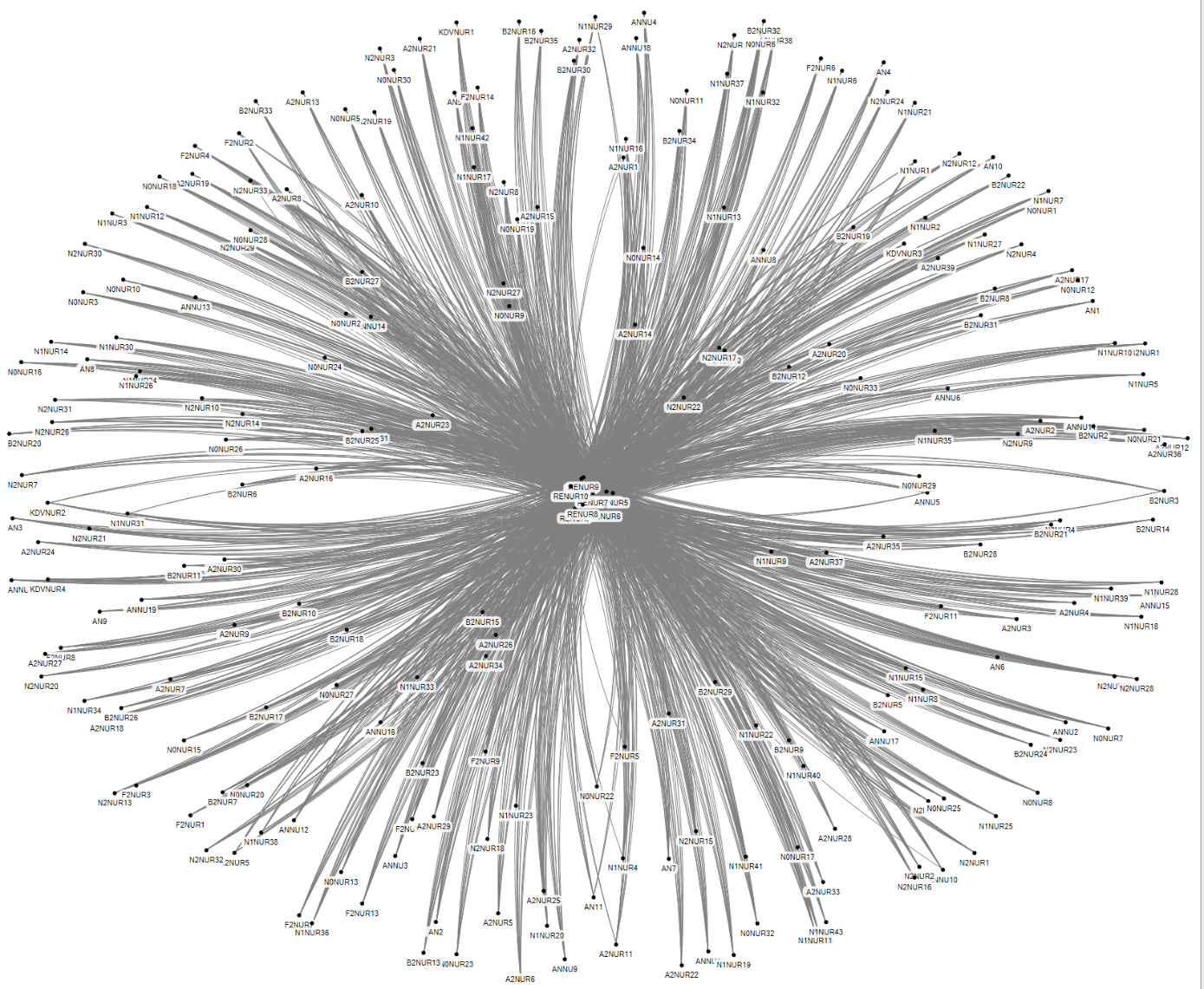


**Figure 20** Social network of task 20: Patient care in recovery

| **Parameter** | **Value** |
| --- | --- |
| Number of agents | 241 |
| Number of unique ties | 2,355 |
| Density | 0.08 |
| Number of cliques | PM |
| Highest betweenness centrality | PM |

After surgery the patient is transferred to recovery by the nurse anesthetist. Again the transfer is performed using the standard transfer protocol. In recovery an image might be made of the surgery result, for which a radiology staff member is called. The recovery nurses interact with anesthesiologists on the medication policy if necessary.

1.
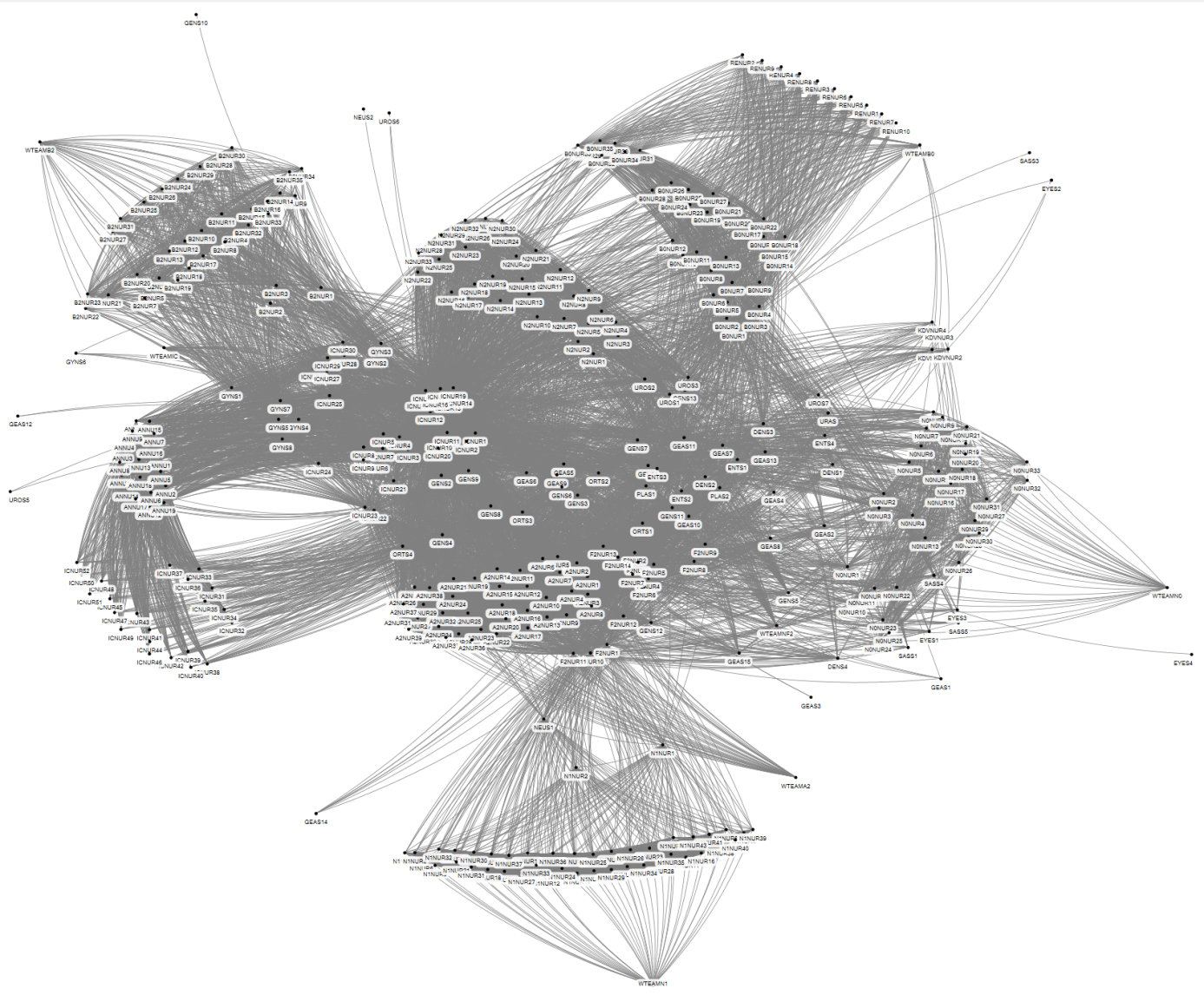
**Aftercare of patient**

**Figure 21** Social network of task 21: Aftercare of patient

| **Parameter** | **Value** |
| --- | --- |
| Number of agents | 391 |
| Number of unique ties | 12,537 |
| Density | 0.16 |
| Number of cliques | 178 |
| Highest betweenness centrality | Nurse 1 ward F2 |

When the patient is completely awake and is not experiencing too much pain in recovery, he is transferred to the nursing ward. The recovery nurse calls the nursing ward to indicate that the patient is to be picked up. The recovery nurse then hands the patient over to the ward nurse, using the standard transfer protocol. The patient is taken care of in one of the nursing wards until he or she is recovered well enough to be discharged. Some patients are transferred to another nursing ward and a transfer from nurses from one department to the nurse of the receiving department takes place. Surgeons visit the patients in the nursing ward and discuss their patients with the nurse and physician responsible for the ward on that day.

1. **Manage the OTC day program**


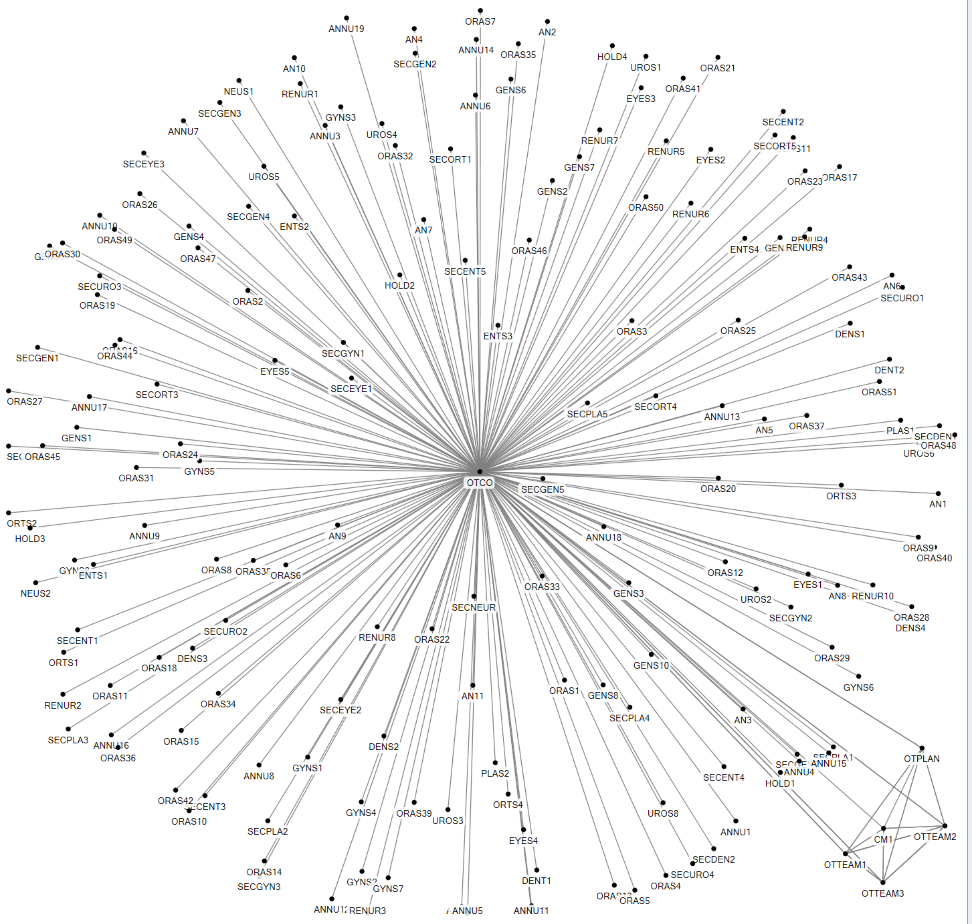


**Figure 22** Social network of task 22: Managing the OTC day program

| **Parameter** | **Value** |
| --- | --- |
| Number of agents | 185 |
| Number of unique ties | 181 |
| Density | 0.01 |
| Number of cliques | 2 |
| Highest betweenness centrality | OTCO |

There are several tasks, performed at various moments in time, aimed at making sure that the surgeries that are planned for one day are executed well and on time. Every day starts with a start of day meeting between the OTC day coordinator and the three OTC team leaders, in which the expectations and special surgeries are discussed.

During the course of the day the day coordinator monitors the progress of each OR, in order to prevent bottlenecks in case surgeries last longer than planned or unexpected events occur, such as emergency patients, failing equipment and such. If the day coordinator foresees that more than two ORs will be running late, he communicates with an anesthesiologist or surgeons about any alterations required in the OR scheme. The anesthesiologist plays a role here, because they work across different ORs throughout the day and they have an interest in the OR scheme being executed as planned. They are used as the intermediate between the day coordinator and the surgeons, who take more interest in completing the entire scheme, taking any overtime or reshuffling for granted. This relationship is included in the task Collaborating in the OR.

1. **Manage OTC tasks**


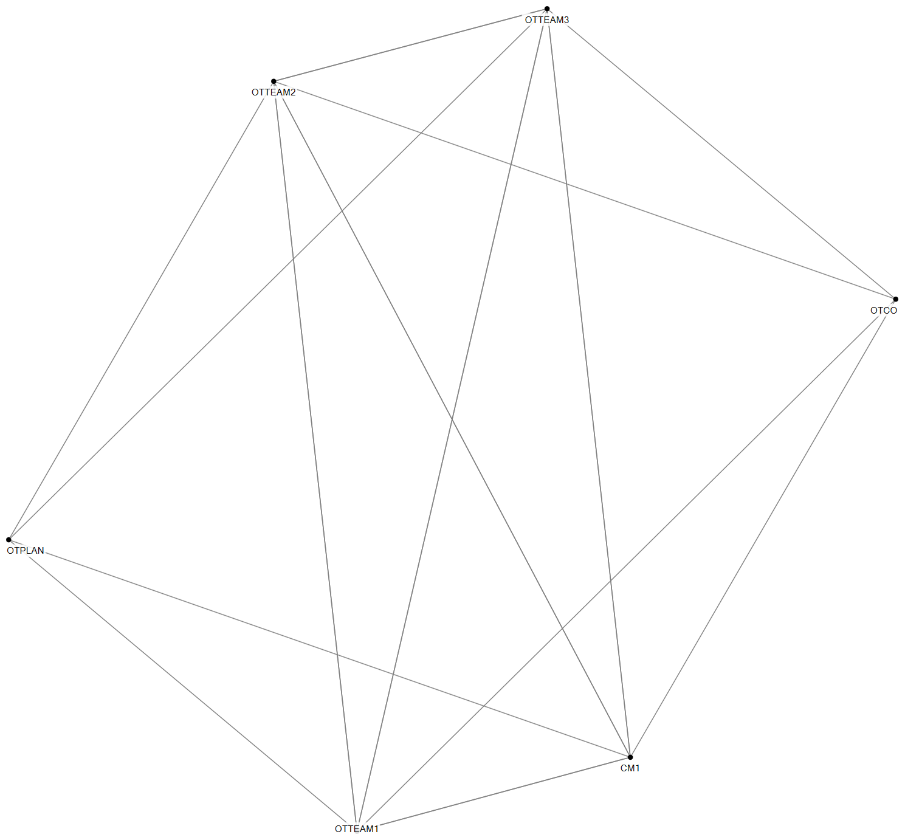


**Figure 23** Social network of task 23: Manage OTC tasks

| **Parameter** | **Value** |
| --- | --- |
| Number of agents | 6 |
| Number of unique ties | 14 |
| Density | 0.93 |
| Number of cliques | 2 |
| Highest betweenness centrality | CM1/ OTTEAM1/ OTTEAM2/ OTTEAM3 |

There are several regular meetings between the OTC cluster manager, OTC team leaders, the OTC capacity planner and the OTC day coordinator to discuss operations over a longer period of time.
