# Supplementary material for: Identifying integration and differentiation in a Hospital’s logistical system: a social network analysis of a case study
Source: BMC Health Serv Res. 2020 Sep 11;20:857. doi: 10.1186/s12913-020-05514-w (PMC7488445; doi:10.1186/s12913-020-05514-w)
Supplement: Supplementary file 3 — Additional file 3. Description and degree of agents in social networks. The code names, names and network degree of all agents are presented in Additional File 3. [file 12913_2020_5514_MOESM3_ESM.docx]

**Additional file 3: Description and degree of agents in social networks**

| **Agent code** | **Description** | **Degree** |
| --- | --- | --- |
| ANNU17 | Nurse anesthetist 17 | 399 |
| ANNU16 | Nurse anesthetist 16 | 393 |
| ANNU1 | Nurse anesthetist 1 | 390 |
| ANNU11 | Nurse anesthetist 11 | 390 |
| ANNU5 | Nurse anesthetist 5 | 389 |
| ANNU12 | Nurse anesthetist 12 | 388 |
| ANNU2 | Nurse anesthetist 1 | 388 |
| ANNU2 | Nurse anesthetist 2 | 388 |
| ANNU10 | Nurse anesthetist 10 | 387 |
| ANNU13 | Nurse anesthetist 13 | 387 |
| ANNU3 | Nurse anesthetist 3 | 387 |
| ANNU15 | Nurse anesthetist 15 | 386 |
| ANNU18 | Nurse anesthetist 18 | 386 |
| ANNU6 | Nurse anesthetist 6 | 386 |
| ANNU7 | Nurse anesthetist 7 | 386 |
| ANNU14 | Nurse anesthetist 14 | 385 |
| ANNU19 | Nurse anesthetist19 | 384 |
| ANNU4 | Nurse anesthetist 4 | 384 |
| ANNU9 | Nurse anesthetist 9 | 384 |
| ANNU8 | Nurse anesthetist 8 | 383 |
| RENUR1 | Recovery nurse 1 | 318 |
| RENUR10 | Recovery nurse 10 | 318 |
| RENUR2 | Recovery nurse 2 | 318 |
| RENUR3 | Recovery nurse 3 | 318 |
| RENUR4 | Recovery nurse 4 | 318 |
| RENUR5 | Recovery nurse 5 | 318 |
| RENUR6 | Recovery nurse 6 | 318 |
| RENUR7 | Recovery nurse 7 | 318 |
| RENUR8 | Recovery nurse 8 | 318 |
| RENUR9 | Recovery nurse 9 | 318 |
| HOLD2 | Holding nurse 2 | 312 |
| HOLD3 | Holding nurse 3 | 312 |
| HOLD4 | Holding nurse 4 | 312 |
| OTCO | OTC day coordinator | 294 |
| HOLD1 | Holding nurse 1 | 266 |
| GENS6 | General surgeon 6 | 263 |
| GEAS6 | Assistant surgeon 6 | 260 |
| GENS1 | General surgeon 1 | 251 |
| GEAS9 | Assistant surgeon 9 | 247 |
| GENS7 | General surgeon 7 | 246 |
| ICNUR1 | Intensive care nurse 1 | 220 |
| GENS9 | General surgeon 9 | 218 |
| GENS3 | General surgeon 3 | 213 |
| GENS8 | General surgeon 8 | 211 |
| GENS2 | General surgeon 2 | 209 |
| ICNUR2 | Intensive care nurse 2 | 209 |
| GENS4 | General surgeon 4 | 203 |
| ORTS2 | Orthopedic surgeon 2 | 203 |
| GEAS5 | Assistant surgeon 5 | 202 |
| ICNUR10 | Intensive care nurse 10 | 202 |
| ICNUR11 | Intensive care nurse 11 | 201 |
| ICNUR12 | Intensive care nurse 12 | 198 |
| F2NUR1 | Ward F2 nurse 1 | 194 |
| ICNUR13 | Intensive care nurse 13 | 192 |
| ICNUR14 | Intensive care nurse 14 | 192 |
| ORTS4 | Orthopedic surgeon 4 | 192 |
| ICNUR15 | Intensive care nurse 15 | 190 |
| GENS11 | General surgeon 11 | 189 |
| F2NUR10 | Ward F2 nurse 10 | 188 |
| PLAS1 | Plastic surgeon 1 | 188 |
| ICNUR16 | Intensive care nurse 16 | 187 |
| ICNUR17 | Intensive care nurse 17 | 187 |
| ORTS3 | Orthopedic surgeon 3 | 187 |
| ICNUR18 | Intensive care nurse 18 | 184 |
| F2NUR11 | Ward F2 nurse 11 | 183 |
| ICNUR19 | Intensive care nurse 19 | 183 |
| ICNUR20 | Intensive care nurse 20 | 182 |
| UROS2 | Urology surgeon2 | 176 |
| ORTS1 | Orthopedic surgeon 1 | 173 |
| ENTS3 | Ear Nose Throat surgeon 3 | 170 |
| UROS1 | Urology surgeon1 | 169 |
| UROS3 | Urology surgeon3 | 169 |
| GEAS10 | Assistant surgeon 10 | 164 |
| ENTS1 | Ear Nose Throat surgeon 1 | 163 |
| ICNUR21 | Intensive care nurse 21 | 163 |
| PLAS2 | Plastic surgeon 2 | 161 |
| GEAS11 | Assistant surgeon 11 | 160 |
| ENTS2 | Ear Nose Throat surgeon 2 | 156 |
| F2NUR12 | Ward F2 nurse 12 | 155 |
| F2NUR2 | Ward F2 nurse 2 | 155 |
| F2NUR3 | Ward F2 nurse 3 | 155 |
| F2NUR4 | Ward F2 nurse 4 | 153 |
| GENS5 | General surgeon 5 | 153 |
| F2NUR5 | Ward F2 nurse 5 | 151 |
| DENS2 | Dental surgeon 2 | 149 |
| F2NUR6 | Ward F2 nurse 6 | 149 |
| F2NUR7 | Ward F2 nurse 7 | 149 |
| ICNUR22 | Intensive care nurse 22 | 147 |
| GENS12 | General surgeon 12 | 146 |
| ICNUR23 | Intensive care nurse 23 | 144 |
| GYNS7 | Gynecological surgeon 7 | 143 |
| GYNS2 | Gynecological surgeon 2 | 141 |
| N0NUR1 | Ward N0 nurse 1 | 141 |
| F2NUR13 | Ward F2 nurse 13 | 140 |
| GYNS4 | Gynecological surgeon 4 | 140 |
| A2NUR1 | Ward A2 nurse 1 | 139 |
| A2NUR2 | Ward A2 nurse 2 | 139 |
| F2NUR14 | Ward F2 nurse 14 | 139 |
| GYNS3 | Gynecological surgeon 3 | 137 |
| N0NUR2 | Ward N0 nurse 2 | 137 |
| A2NUR3 | Ward A2 nurse 3 | 136 |
| A2NUR4 | Ward A2 nurse 4 | 136 |
| DENS3 | Dental surgeon 3 | 136 |
| N0NUR3 | Ward N0 nurse 3 | 136 |
| A2NUR5 | Ward A2 nurse 5 | 135 |
| A2NUR6 | Ward A2 nurse 6 | 135 |
| A2NUR7 | Ward A2 nurse 7 | 135 |
| GENS13 | General surgeon 13 | 135 |
| GYNS5 | Gynecological surgeon 5 | 135 |
| ICNUR3 | Intensive care nurse 3 | 135 |
| ORAS17 | OR nurse 17 | 135 |
| GEAS7 | Assistant surgeon 7 | 134 |
| ORAS1 | OR nurse 1 | 134 |
| ICNUR4 | Intensive care nurse 4 | 133 |
| A2NUR8 | Ward A2 nurse 8 | 132 |
| A2NUR10 | Ward A2 nurse 10 | 131 |
| A2NUR11 | Ward A2 nurse 11 | 131 |
| ORAS11 | OR nurse 11 | 131 |
| ORAS40 | OR nurse 40 | 131 |
| GYNS1 | Gynecological surgeon 1 | 130 |
| ICNUR5 | Intensive care nurse 5 | 130 |
| ORAS23 | OR nurse 23 | 130 |
| A2NUR12 | Ward A2 nurse 12 | 129 |
| A2NUR9 | Ward A2 nurse 9 | 129 |
| GYNS8 | Gynecological surgeon 8 | 129 |
| ICNUR6 | Intensive care nurse 6 | 129 |
| ICNUR7 | Intensive care nurse 7 | 129 |
| ORAS27 | OR nurse 27 | 129 |
| ORAS3 | OR nurse 3 | 129 |
| A2NUR13 | Ward A2 nurse 13 | 128 |
| ORAS13 | OR nurse 13 | 128 |
| ORAS29 | OR nurse 29 | 128 |
| A2NUR14 | Ward A2 nurse 14 | 127 |
| A2NUR15 | Ward A2 nurse 15 | 127 |
| A2NUR16 | Ward A2 nurse 16 | 127 |
| ORAS18 | OR nurse 18 | 127 |
| ORAS19 | OR nurse 19 | 127 |
| ORAS21 | OR nurse 21 | 127 |
| ORAS10 | OR nurse 10 | 126 |
| ORAS16 | OR nurse 16 | 126 |
| A2NUR17 | Ward A2 nurse 17 | 125 |
| A2NUR18 | Ward A2 nurse 18 | 125 |
| ICNUR8 | Intensive care nurse 8 | 125 |
| ICNUR9 | Intensive care nurse 9 | 125 |
| ORAS2 | OR nurse 2 | 125 |
| ORAS20 | OR nurse 20 | 125 |
| ORAS28 | OR nurse 28 | 125 |
| ORAS36 | OR nurse 36 | 125 |
| A2NUR19 | Ward A2 nurse 19 | 124 |
| A2NUR20 | Ward A2 nurse 20 | 124 |
| ORAS12 | OR nurse 12 | 124 |
| ORAS14 | OR nurse 14 | 124 |
| ORAS30 | OR nurse 30 | 124 |
| ORAS31 | OR nurse 31 | 124 |
| F2NUR8 | Ward F2 nurse 8 | 123 |
| NEUS1 | Neurosurgeon 1 | 123 |
| ORAS24 | OR nurse 24 | 123 |
| ORAS38 | OR nurse 38 | 123 |
| ORAS47 | OR nurse 47 | 123 |
| A2NUR21 | Ward A2 nurse 21 | 122 |
| B0NUR1 | Ward B0 nurse 1 | 122 |
| A2NUR22 | Ward A2 nurse 22 | 121 |
| GEAS13 | Assistant surgeon 13 | 121 |
| ORAS32 | OR nurse 32 | 121 |
| ORAS34 | OR nurse 34 | 121 |
| A2NUR23 | Ward A2 nurse 23 | 120 |
| A2NUR24 | Ward A2 nurse 24 | 120 |
| A2NUR25 | Ward A2 nurse 25 | 120 |
| ORAS22 | OR nurse 22 | 120 |
| ORAS4 | OR nurse 4 | 120 |
| ORAS42 | OR nurse 42 | 120 |
| A2NUR26 | Ward A2 nurse 26 | 119 |
| A2NUR27 | Ward A2 nurse 27 | 119 |
| A2NUR28 | Ward A2 nurse 28 | 119 |
| A2NUR29 | Ward A2 nurse 29 | 119 |
| A2NUR32 | Ward A2 nurse 32 | 119 |
| A2NUR33 | Ward A2 nurse 33 | 119 |
| A2NUR34 | Ward A2 nurse 34 | 119 |
| A2NUR35 | Ward A2 nurse 35 | 119 |
| A2NUR36 | Ward A2 nurse 36 | 119 |
| A2NUR37 | Ward A2 nurse 37 | 119 |
| A2NUR38 | Ward A2 nurse 38 | 119 |
| ORAS26 | OR nurse 26 | 119 |
| ORAS33 | OR nurse 33 | 119 |
| ORAS37 | OR nurse 37 | 119 |
| ORAS39 | OR nurse 39 | 119 |
| ORAS46 | OR nurse 46 | 119 |
| A2NUR30 | Ward A2 nurse 30 | 118 |
| A2NUR31 | Ward A2 nurse 31 | 118 |
| ORAS51 | OR nurse 51 | 118 |
| A2NUR39 | Ward A2 nurse 39 | 117 |
| N0NUR10 | Ward N0 nurse 10 | 117 |
| ORAS25 | OR nurse 25 | 117 |
| ORAS35 | OR nurse 35 | 117 |
| ORAS45 | OR nurse 45 | 117 |
| ORAS48 | OR nurse 48 | 117 |
| ORAS49 | OR nurse 49 | 117 |
| ORAS7 | OR nurse 7 | 117 |
| AN1 | Anesthesiologist 1 | 116 |
| N0NUR11 | Ward N0 nurse 11 | 116 |
| AN11 | Anesthesiologist 11 | 115 |
| B0NUR2 | Ward B0 nurse 2 | 115 |
| GEAS15 | Assistant surgeon 15 | 114 |
| N0NUR12 | Ward N0 nurse 12 | 114 |
| N2NUR1 | Ward N2 nurse 1 | 113 |
| ORAS41 | OR nurse 41 | 113 |
| AN10 | Anesthesiologist 10 | 112 |
| AN2 | Anesthesiologist 2 | 112 |
| AN4 | Anesthesiologist 4 | 112 |
| AN7 | Anesthesiologist 7 | 112 |
| AN8 | Anesthesiologist 8 | 112 |
| ORAS43 | OR nurse 43 | 112 |
| AN3 | Anesthesiologist 3 | 111 |
| AN6 | Anesthesiologist 6 | 111 |
| GEAS8 | Assistant surgeon 8 | 111 |
| ORAS44 | OR nurse 44 | 111 |
| ORAS6 | OR nurse 6 | 111 |
| AN5 | Anesthesiologist 5 | 110 |
| AN9 | Anesthesiologist 9 | 110 |
| B0NUR3 | Ward B0 nurse 3 | 110 |
| N0NUR13 | Ward N0 nurse 13 | 110 |
| N0NUR14 | Ward N0 nurse 14 | 110 |
| N0NUR15 | Ward N0 nurse 15 | 110 |
| N0NUR16 | Ward N0 nurse 16 | 109 |
| F2NUR9 | Ward F2 nurse 9 | 108 |
| N0NUR17 | Ward N0 nurse 17 | 108 |
| OTPLAN | OTC capacity planner | 108 |
| N0NUR18 | Ward N0 nurse 18 | 107 |
| N0NUR19 | Ward N0 nurse 19 | 107 |
| N0NUR20 | Ward N0 nurse 20 | 107 |
| N0NUR21 | Ward N0 nurse 21 | 107 |
| N0NUR22 | Ward N0 nurse 22 | 107 |
| ORAS50 | OR nurse 50 | 107 |
| ICNUR24 | Intensive care nurse 24 | 106 |
| ICNUR25 | Intensive care nurse 25 | 106 |
| N0NUR23 | Ward N0 nurse 23 | 106 |
| N2NUR2 | Ward N2 nurse 2 | 106 |
| ICNUR26 | Intensive care nurse 26 | 105 |
| ICNUR27 | Intensive care nurse 27 | 105 |
| ICNUR28 | Intensive care nurse 28 | 105 |
| ICNUR29 | Intensive care nurse 29 | 105 |
| ICNUR30 | Intensive care nurse 30 | 105 |
| N0NUR24 | Ward N0 nurse 24 | 105 |
| N0NUR25 | Ward N0 nurse 25 | 105 |
| N0NUR26 | Ward N0 nurse 26 | 105 |
| N0NUR27 | Ward N0 nurse 27 | 105 |
| N0NUR28 | Ward N0 nurse 28 | 105 |
| N0NUR29 | Ward N0 nurse 29 | 105 |
| N0NUR30 | Ward N0 nurse 30 | 105 |
| N0NUR31 | Ward N0 nurse 31 | 105 |
| B0NUR4 | Ward B0 nurse 4 | 104 |
| N2NUR3 | Ward N2 nurse 3 | 104 |
| B0NUR5 | Ward B0 nurse 5 | 103 |
| N0NUR32 | Ward N0 nurse 32 | 103 |
| N0NUR33 | Ward N0 nurse 33 | 103 |
| ORAS5 | OR nurse 5 | 103 |
| N2NUR4 | Ward N2 nurse 4 | 102 |
| N2NUR5 | Ward N2 nurse 5 | 102 |
| B0NUR6 | Ward B0 nurse 6 | 101 |
| N2NUR6 | Ward N2 nurse 6 | 101 |
| N2NUR7 | Ward N2 nurse 7 | 100 |
| B2NUR1 | Ward B2 nurse 1 | 99 |
| DENS1 | Dental surgeon 1 | 98 |
| GEAS4 | Assistant surgeon 4 | 98 |
| N2NUR10 | Ward N2 nurse 10 | 98 |
| N2NUR8 | Ward N2 nurse 8 | 97 |
| N2NUR9 | Ward N2 nurse 9 | 97 |
| B0NUR7 | Ward B0 nurse 7 | 95 |
| N0NUR4 | Ward N0 nurse 4 | 94 |
| B0NUR8 | Ward B0 nurse 8 | 93 |
| N0NUR5 | Ward N0 nurse 5 | 93 |
| N2NUR11 | Ward N2 nurse 11 | 93 |
| N2NUR12 | Ward N2 nurse 12 | 93 |
| N2NUR13 | Ward N2 nurse 13 | 93 |
| B0NUR9 | Ward B0 nurse 9 | 92 |
| N2NUR14 | Ward N2 nurse 14 | 92 |
| N2NUR15 | Ward N2 nurse 15 | 92 |
| N2NUR16 | Ward N2 nurse 16 | 91 |
| N2NUR17 | Ward N2 nurse 17 | 91 |
| N2NUR18 | Ward N2 nurse 18 | 91 |
| N2NUR19 | Ward N2 nurse 19 | 91 |
| B0NUR10 | Ward B0 nurse 10 | 89 |
| B2NUR2 | Ward B2 nurse 2 | 89 |
| ENTS4 | Ear Nose Throat surgeon 4 | 89 |
| ICNUR31 | Intensive care nurse 31 | 89 |
| ICNUR32 | Intensive care nurse 32 | 89 |
| ICNUR33 | Intensive care nurse 33 | 89 |
| ICNUR34 | Intensive care nurse 34 | 89 |
| N0NUR6 | Ward N0 nurse 6 | 89 |
| N2NUR20 | Ward N2 nurse 20 | 89 |
| N2NUR21 | Ward N2 nurse 21 | 89 |
| ICNUR36 | Intensive care nurse 36 | 88 |
| N2NUR22 | Ward N2 nurse 22 | 88 |
| B0NUR11 | Ward B0 nurse 11 | 87 |
| B0NUR12 | Ward B0 nurse 12 | 87 |
| DENS4 | Dental surgeon 4 | 87 |
| ICNUR35 | Intensive care nurse 35 | 87 |
| ICNUR37 | Intensive care nurse 37 | 87 |
| N0NUR7 | Ward N0 nurse 7 | 87 |
| N2NUR23 | Ward N2 nurse 23 | 87 |
| N2NUR24 | Ward N2 nurse 24 | 87 |
| N2NUR25 | Ward N2 nurse 25 | 87 |
| B0NUR13 | Ward B0 nurse 13 | 86 |
| ICNUR38 | Intensive care nurse 38 | 86 |
| N2NUR26 | Ward N2 nurse 26 | 86 |
| N2NUR27 | Ward N2 nurse 27 | 86 |
| N2NUR28 | Ward N2 nurse 28 | 86 |
| N2NUR29 | Ward N2 nurse 29 | 86 |
| N2NUR30 | Ward N2 nurse 30 | 86 |
| N2NUR31 | Ward N2 nurse 31 | 86 |
| N2NUR32 | Ward N2 nurse 32 | 86 |
| N2NUR33 | Ward N2 nurse 33 | 86 |
| ICNUR39 | Intensive care nurse 39 | 85 |
| N1NUR1 | Ward N1 nurse 1 | 85 |
| ORAS15 | OR nurse 15 | 85 |
| B2NUR3 | Ward B2 nurse 3 | 84 |
| ICNUR40 | Intensive care nurse 40 | 84 |
| N0NUR8 | Ward N0 nurse 8 | 84 |
| B0NUR14 | Ward B0 nurse 14 | 83 |
| ICNUR41 | Intensive care nurse 41 | 83 |
| ICNUR42 | Intensive care nurse 42 | 83 |
| ICNUR43 | Intensive care nurse 43 | 83 |
| N0NUR9 | Ward N0 nurse 9 | 83 |
| B2NUR10 | Ward B2 nurse 10 | 82 |
| B2NUR11 | Ward B2 nurse 11 | 82 |
| B2NUR12 | Ward B2 nurse 12 | 82 |
| B2NUR13 | Ward B2 nurse 13 | 82 |
| B2NUR14 | Ward B2 nurse 14 | 82 |
| B2NUR15 | Ward B2 nurse 15 | 82 |
| B2NUR16 | Ward B2 nurse 16 | 82 |
| B2NUR17 | Ward B2 nurse 17 | 82 |
| B2NUR18 | Ward B2 nurse 18 | 82 |
| B2NUR19 | Ward B2 nurse 19 | 82 |
| B2NUR20 | Ward B2 nurse 20 | 82 |
| B2NUR21 | Ward B2 nurse 21 | 82 |
| B2NUR22 | Ward B2 nurse 22 | 82 |
| B2NUR23 | Ward B2 nurse 23 | 82 |
| B2NUR24 | Ward B2 nurse 24 | 82 |
| B2NUR25 | Ward B2 nurse 25 | 82 |
| B2NUR26 | Ward B2 nurse 26 | 82 |
| B2NUR27 | Ward B2 nurse 27 | 82 |
| ICNUR44 | Intensive care nurse 44 | 82 |
| N1NUR2 | Ward N1 nurse 2 | 82 |
| B0NUR15 | Ward B0 nurse 15 | 81 |
| B2NUR28 | Ward B2 nurse 28 | 81 |
| B2NUR29 | Ward B2 nurse 29 | 81 |
| B2NUR9 | Ward B2 nurse 9 | 81 |
| ICNUR45 | Intensive care nurse 45 | 81 |
| ICNUR46 | Intensive care nurse 46 | 81 |
| ICNUR47 | Intensive care nurse 47 | 81 |
| ICNUR48 | Intensive care nurse 48 | 81 |
| B2NUR30 | Ward B2 nurse 30 | 80 |
| B2NUR31 | Ward B2 nurse 31 | 80 |
| B2NUR32 | Ward B2 nurse 32 | 80 |
| B2NUR33 | Ward B2 nurse 33 | 80 |
| B2NUR4 | Ward B2 nurse 4 | 80 |
| B2NUR8 | Ward B2 nurse 8 | 80 |
| ERPHYS1 | ER physician 1 | 80 |
| ERPHYS4 | ER physician 4 | 80 |
| ERPHYS5 | ER physician 5 | 80 |
| ERPHYS6 | ER physician 6 | 80 |
| ICNUR49 | Intensive care nurse 49 | 80 |
| N1NUR10 | Ward N1 nurse 10 | 80 |
| N1NUR11 | Ward N1 nurse 11 | 80 |
| N1NUR12 | Ward N1 nurse 12 | 80 |
| N1NUR13 | Ward N1 nurse 13 | 80 |
| N1NUR14 | Ward N1 nurse 14 | 80 |
| N1NUR15 | Ward N1 nurse 15 | 80 |
| N1NUR16 | Ward N1 nurse 16 | 80 |
| N1NUR17 | Ward N1 nurse 17 | 80 |
| N1NUR18 | Ward N1 nurse 18 | 80 |
| N1NUR19 | Ward N1 nurse 19 | 80 |
| N1NUR20 | Ward N1 nurse 20 | 80 |
| N1NUR21 | Ward N1 nurse 21 | 80 |
| N1NUR22 | Ward N1 nurse 22 | 80 |
| N1NUR23 | Ward N1 nurse 23 | 80 |
| N1NUR24 | Ward N1 nurse 24 | 80 |
| N1NUR25 | Ward N1 nurse 25 | 80 |
| N1NUR26 | Ward N1 nurse 26 | 80 |
| N1NUR27 | Ward N1 nurse 27 | 80 |
| N1NUR28 | Ward N1 nurse 28 | 80 |
| N1NUR29 | Ward N1 nurse 29 | 80 |
| N1NUR3 | Ward N1 nurse 3 | 80 |
| N1NUR30 | Ward N1 nurse 30 | 80 |
| N1NUR31 | Ward N1 nurse 31 | 80 |
| N1NUR32 | Ward N1 nurse 32 | 80 |
| N1NUR33 | Ward N1 nurse 33 | 80 |
| N1NUR34 | Ward N1 nurse 34 | 80 |
| N1NUR35 | Ward N1 nurse 35 | 80 |
| N1NUR36 | Ward N1 nurse 36 | 80 |
| N1NUR37 | Ward N1 nurse 37 | 80 |
| N1NUR38 | Ward N1 nurse 38 | 80 |
| N1NUR39 | Ward N1 nurse 39 | 80 |
| N1NUR4 | Ward N1 nurse 4 | 80 |
| N1NUR40 | Ward N1 nurse 40 | 80 |
| N1NUR41 | Ward N1 nurse 41 | 80 |
| N1NUR42 | Ward N1 nurse 42 | 80 |
| N1NUR43 | Ward N1 nurse 43 | 80 |
| N1NUR5 | Ward N1 nurse 5 | 80 |
| N1NUR6 | Ward N1 nurse 6 | 80 |
| N1NUR7 | Ward N1 nurse 7 | 80 |
| N1NUR8 | Ward N1 nurse 8 | 80 |
| N1NUR9 | Ward N1 nurse 9 | 80 |
| B0NUR16 | Ward B0 nurse 16 | 79 |
| B0NUR17 | Ward B0 nurse 17 | 79 |
| B2NUR5 | Ward B2 nurse 5 | 79 |
| B2NUR6 | Ward B2 nurse 6 | 79 |
| B2NUR7 | Ward B2 nurse 7 | 79 |
| ICNUR50 | Intensive care nurse 50 | 79 |
| ICNUR51 | Intensive care nurse 51 | 79 |
| B0NUR18 | Ward B0 nurse 18 | 78 |
| ICNUR52 | Intensive care nurse 52 | 78 |
| B0NUR19 | Ward B0 nurse 19 | 77 |
| B0NUR20 | Ward B0 nurse 20 | 77 |
| ERPHYS3 | ER physician 3 | 77 |
| B0NUR21 | Ward B0 nurse 21 | 76 |
| B0NUR22 | Ward B0 nurse 22 | 76 |
| B0NUR23 | Ward B0 nurse 23 | 76 |
| B0NUR24 | Ward B0 nurse 24 | 76 |
| ERPHYS2 | ER physician 2 | 76 |
| B0NUR25 | Ward B0 nurse 25 | 75 |
| B0NUR26 | Ward B0 nurse 26 | 75 |
| B0NUR27 | Ward B0 nurse 27 | 75 |
| B0NUR28 | Ward B0 nurse 28 | 75 |
| B0NUR29 | Ward B0 nurse 29 | 74 |
| B0NUR30 | Ward B0 nurse 30 | 74 |
| B0NUR31 | Ward B0 nurse 31 | 74 |
| B0NUR32 | Ward B0 nurse 32 | 74 |
| B0NUR33 | Ward B0 nurse 33 | 74 |
| B0NUR34 | Ward B0 nurse 34 | 74 |
| B0NUR35 | Ward B0 nurse 35 | 74 |
| B2NUR34 | Ward B2 nurse 34 | 74 |
| B2NUR35 | Ward B2 nurse 35 | 74 |
| ORAS8 | OR nurse 8 | 74 |
| ERNUR1 | ER nurse 1 | 68 |
| ERNUR10 | ER nurse 10 | 68 |
| ERNUR11 | ER nurse 11 | 68 |
| ERNUR12 | ER nurse 12 | 68 |
| ERNUR13 | ER nurse 13 | 68 |
| ERNUR14 | ER nurse 14 | 68 |
| ERNUR15 | ER nurse 15 | 68 |
| ERNUR16 | ER nurse 16 | 68 |
| ERNUR17 | ER nurse 17 | 68 |
| ERNUR18 | ER nurse 18 | 68 |
| ERNUR19 | ER nurse 19 | 68 |
| ERNUR2 | ER nurse 2 | 68 |
| ERNUR20 | ER nurse 20 | 68 |
| ERNUR21 | ER nurse 21 | 68 |
| ERNUR22 | ER nurse 22 | 68 |
| ERNUR23 | ER nurse 23 | 68 |
| ERNUR24 | ER nurse 24 | 68 |
| ERNUR25 | ER nurse 25 | 68 |
| ERNUR26 | ER nurse 26 | 68 |
| ERNUR27 | ER nurse 27 | 68 |
| ERNUR28 | ER nurse 28 | 68 |
| ERNUR29 | ER nurse 29 | 68 |
| ERNUR3 | ER nurse 3 | 68 |
| ERNUR30 | ER nurse 30 | 68 |
| ERNUR31 | ER nurse 31 | 68 |
| ERNUR32 | ER nurse 32 | 68 |
| ERNUR33 | ER nurse 33 | 68 |
| ERNUR34 | ER nurse 34 | 68 |
| ERNUR35 | ER nurse 35 | 68 |
| ERNUR4 | ER nurse 4 | 68 |
| ERNUR5 | ER nurse 5 | 68 |
| ERNUR6 | ER nurse 6 | 68 |
| ERNUR7 | ER nurse 7 | 68 |
| ERNUR8 | ER nurse 8 | 68 |
| ERNUR9 | ER nurse 9 | 68 |
| EYES1 | Eye surgeon1 | 68 |
| ORAS9 | OR nurse 9 | 67 |
| SECANE1 | Secretary1 Anesthesia | 67 |
| SECANE2 | Secretary2 Anesthesia | 67 |
| SECANE3 | Secretary3 Anesthesia | 67 |
| SECANE4 | Secretary4 Anesthesia | 67 |
| GEAS14 | Assistant surgeon 14 | 65 |
| WTEAMA2 | Ward team leader A2 | 65 |
| UROS7 | Urology surgeon7 | 63 |
| WTEAMNF2 | Ward team leader F2 | 63 |
| KDVNUR1 | Pediatric ward nurse 1 | 60 |
| URAS | Urology assistant surgeon | 60 |
| GEAS2 | Assistant surgeon 2 | 59 |
| KDVNUR2 | Pediatric ward nurse 2 | 57 |
| OTTEAM1 | Team leader Surgery | 57 |
| KDVNUR3 | Pediatric ward nurse 3 | 54 |
| KDVNUR4 | Pediatric ward nurse 4 | 54 |
| OTLOG1 | OTC logistical staff member 1 | 54 |
| OTLOG2 | OTC logistical staff member 1 | 54 |
| CLEAN1 | Cleaning staff member 1 | 51 |
| CLEAN2 | Cleaning staff member 2 | 51 |
| WTEAMIC | Ward team leader Intensive Care | 50 |
| EYES3 | Eye surgeon 3 | 49 |
| GYNS6 | Gynecology surgeon 6 | 47 |
| AN12 | Anesthesiologist 12 | 46 |
| GENS10 | General surgeon 10 | 46 |
| SASS4 | Physician assistant 4 | 44 |
| UROS5 | Urology surgeon5 | 44 |
| WTEAMN1 | Ward team leader N1 | 43 |
| OTSEC | OTC secretary | 42 |
| GEAS1 | Assistant surgeon 1 | 39 |
| SECNEUR | Secretary of Neurology outpatient department | 39 |
| UROS6 | Urology surgeon 6 | 39 |
| WTEAMB0 | Ward team leader B0 | 39 |
| SECCAR | Secretary of Cardiology outpatient department | 38 |
| GEAS12 | Assistant surgeon 12 | 37 |
| GEAS3 | Assistant surgeon 3 | 36 |
| WTEAMB2 | Ward team leader B2 | 35 |
| UROS4 | Urology surgeon4 | 34 |
| WTEAMN0 | Ward team leader N0 | 33 |
| SASS1 | Physician assistant 1 | 32 |
| UROS8 | Urology surgeon 8 | 31 |
| SECGEN1 | Secretary 1 General surgery | 28 |
| SECGEN2 | Secretary 2 General surgery | 28 |
| SECGEN3 | Secretary 3 General surgery | 28 |
| SECGEN4 | Secretary 4 General surgery | 25 |
| SECGEN5 | Secretary 5 General surgery | 25 |
| SECURO1 | Secretary 1 Urology outpatient department | 25 |
| EYES2 | Eye surgeon 2 | 24 |
| OTTEAM2 | Team leader Anesthesia | 24 |
| SECGEN6 | Secretary 6 General surgery | 24 |
| SECGYN1 | Secretary 1 Gynecology | 24 |
| SECGYN2 | Secretary 2 Gynecology | 24 |
| SECGYN3 | Secretary 3 Gynecology | 24 |
| SECORT1 | Secretary 1 Orthopedics outpatient department | 24 |
| SASS5 | Physician assistant 5 | 22 |
| SECURO2 | Secretary 2 Urology outpatient department | 22 |
| SECURO3 | Secretary 3 Urology outpatient department | 22 |
| SECURO4 | Secretary 4 Urology outpatient department | 22 |
| SECORT2 | Secretary 2 Orthopedics outpatient department | 21 |
| SECORT3 | Secretary 3 Orthopedics outpatient department | 21 |
| SECORT4 | Secretary 4 Orthopedics outpatient department | 21 |
| SECORT5 | Secretary 5 Orthopedics outpatient department | 21 |
| SECENT1 | Secretary1 ENT | 20 |
| OTTEAM3 | Team leader Recovery/holding | 19 |
| SECEYE1 | Secretary 1 Ophthalmology | 19 |
| CM1 | Cluster manager OTC and Services | 18 |
| SECDEN1 | Secretary1 Dental Surgery | 18 |
| SECPLA1 | Secretary1 Plastic surgery outpatient department | 18 |
| SECENT2 | Secretary2 ENT | 17 |
| SECENT3 | Secretary3 ENT | 17 |
| SECENT4 | Secretary4 ENT | 17 |
| SECENT5 | Secretary5 ENT | 17 |
| EYES4 | Eye surgeon4 | 16 |
| SECEYE2 | Secretary2 Ophthalmology | 16 |
| SECEYE3 | Secretary3 Ophthalmology | 16 |
| POSNUR1 | Preoperative nurse 1 | 15 |
| POSNUR2 | Preoperative nurse 2 | 15 |
| POSNUR2 | Preoperative nurse 3 | 15 |
| POSNUR3 | Preoperative nurse 4 | 15 |
| POSNUR4 | Preoperative nurse 5 | 15 |
| POSNUR5 | Preoperative nurse 6 | 15 |
| SECCLB | Clinical plan boss | 15 |
| SECDEN2 | Secretary2 Dental Surgery | 15 |
| SECPLA2 | Secretary2 Plastic surgery outpatient department | 15 |
| SECPLA3 | Secretary3 Plastic surgery outpatient department | 15 |
| SECPLA4 | Secretary4 Plastic surgery outpatient department | 15 |
| SECPLA5 | Secretary5 Plastic surgery outpatient department | 15 |
| CM2 | Cluster manager Surgery | 14 |
| WTEAM3 | Team leader in training | 13 |
| RADI1 | Radiology staff member 1 | 12 |
| RADI10 | Radiology staff member 10 | 12 |
| RADI11 | Radiology staff member 11 | 12 |
| RADI13 | Radiology staff member 13 | 12 |
| RADI14 | Radiology staff member 14 | 12 |
| RADI15 | Radiology staff member 15 | 12 |
| RADI16 | Radiology staff member 16 | 12 |
| RADI17 | Radiology staff member 17 | 12 |
| RADI18 | Radiology staff member 18 | 12 |
| RADI19 | Radiology staff member 19 | 12 |
| RADI2 | Radiology staff member 2 | 12 |
| RADI20 | Radiology staff member 20 | 12 |
| RADI21 | Radiology staff member 21 | 12 |
| RADI22 | Radiology staff member 22 | 12 |
| RADI23 | Radiology staff member 23 | 12 |
| RADI24 | Radiology staff member 24 | 12 |
| RADI25 | Radiology staff member 25 | 12 |
| RADI26 | Radiology staff member 26 | 12 |
| RADI27 | Radiology staff member 27 | 12 |
| RADI28 | Radiology staff member 28 | 12 |
| RADI29 | Radiology staff member 29 | 12 |
| RADI3 | Radiology staff member 3 | 12 |
| RADI30 | Radiology staff member 30 | 12 |
| RADI31 | Radiology staff member 31 | 12 |
| RADI32 | Radiology staff member 32 | 12 |
| RADI33 | Radiology staff member 33 | 12 |
| RADI34 | Radiology staff member 34 | 12 |
| RADI35 | Radiology staff member 35 | 12 |
| RADI36 | Radiology staff member 36 | 12 |
| RADI37 | Radiology staff member 37 | 12 |
| RADI38 | Radiology staff member 38 | 12 |
| RADI39 | Radiology staff member 39 | 12 |
| RADI4 | Radiology staff member 4 | 12 |
| RADI40 | Radiology staff member 40 | 12 |
| RADI41 | Radiology staff member 41 | 12 |
| RADI5 | Radiology staff member 5 | 12 |
| RADI6 | Radiology staff member 6 | 12 |
| RADI7 | Radiology staff member 7 | 12 |
| RADI8 | Radiology staff member 8 | 12 |
| RADI9 | Radiology staff member 9 | 12 |
| RADI12 | Radiology staff member 12 | 11 |
| SECNEU | Secretary Neurosurgery | 11 |
| EYES5 | Eye surgeon 5 | 9 |
| NEUS2 | Neuro surgeon 2 | 9 |
| CM3 | Cluster manager Women and Child | 6 |
| FARM1 | Pharmacy assistant 1 | 4 |
| FARM2 | Pharmacy assistant 2 | 4 |
| FARM3 | Pharmacy assistant 3 | 4 |
| FARM4 | Pharmacy assistant 4 | 4 |
| FARM5 | Pharmacy assistant 5 | 4 |
| FARM6 | Pharmacy assistant 6 | 4 |
| FARM7 | Pharmacy assistant 7 | 4 |
| SECGER | Secretary of Geriatric outpatient department | 4 |
| SECINT | Secretary of Internal medicine department | 4 |
| SECLAB | Secretary of Laboratory | 4 |
| SECLUN | Secretary of Lung disease outpatient department | 4 |
| SECRAD | Secretary of Radiology | 4 |
| TECH | Technical staff member | 3 |
| CSD1 | Central Sterilization Department staff member 1 | 1 |
| CSD10 | Central Sterilization Department staff member 10 | 1 |
| CSD11 | Central Sterilization Department staff member 11 | 1 |
| CSD12 | Central Sterilization Department staff member 12 | 1 |
| CSD13 | Central Sterilization Department staff member 13 | 1 |
| CSD14 | Central Sterilization Department staff member 14 | 1 |
| CSD15 | Central Sterilization Department staff member 15 | 1 |
| CSD16 | Central Sterilization Department staff member 16 | 1 |
| CSD17 | Central Sterilization Department staff member 17 | 1 |
| CSD18 | Central Sterilization Department staff member 18 | 1 |
| CSD2 | Central Sterilization Department staff member 2 | 1 |
| CSD3 | Central Sterilization Department staff member 3 | 1 |
| CSD4 | Central Sterilization Department staff member 4 | 1 |
| CSD5 | Central Sterilization Department staff member 5 | 1 |
| CSD6 | Central Sterilization Department staff member 6 | 1 |
| CSD7 | Central Sterilization Department staff member 7 | 1 |
| CSD8 | Central Sterilization Department staff member 8 | 1 |
| CSD9 | Central Sterilization Department staff member 9 | 1 |
| SASS3 | Physician assistant 3 | 1 |
| SECWIN | Secretary Winterswijk Surgery outpatient department | 1 |
